# Knowledge and Misinformation Regarding Autism Risk Factors in a Survey of US Adults

**DOI:** 10.1001/jamahealthforum.2026.4105

**Published:** 2026-11-06

**Authors:** Roy H. Perlis, Mauricio Santillana, Matthew A. Baum, James N. Druckman, Katherine Ognyanova, David Lazer

**Affiliations:** 1 Center for Quantitative Health, Massachusetts General Hospital, Boston; 2 Department of Psychiatry, Harvard Medical School, Boston, Massachusetts; 3 Network Science Institute, Northeastern University, Boston, Massachusetts; 4 Institute for Quantitative Social Science, Harvard University, Boston, Massachusetts; 5 John F. Kennedy School of Government, Harvard University, Cambridge, Massachusetts; 6 Department of Government, Harvard University, Cambridge, Massachusetts; 7 Department of Political Science, University of Rochester, Rochester, New York; 8 Department of Communication, School of Communication and Information, Rutgers University, New Brunswick, New Jersey

## Abstract

**Importance:**

With the rise of misinformation about autism risk, including recent statements from US government officials, the extent to which the public understands environmental autism risk factors is not known.

**Objective:**

To characterize beliefs about autism risk factors among US adults and identify characteristics influencing these beliefs.

**Design, Setting, and Participants:**

This cross-sectional survey study used data from an internet-based survey of adults aged 18 years or older residing in all 50 US states and the District of Columbia and was administered between December 18, 2025, and January 27, 2026. Multiple regression models assessed sociodemographic and other individual characteristics, including political affiliation and trust in institutions, for association with endorsement of misinformed beliefs.

**Main Outcomes and Measures:**

The primary outcome was endorsement of individual risk factors, scored on a 5-point Likert scale from definitely false to definitely true.

**Results:**

Of the 30 388 adults who completed the survey, 100 did not provide an answer to the childhood vaccination item, leaving 30 288 in the analysis. Among the 30 288 participants (mean [SD] age, 46.6 [15.9] years), 13 702 (45.2%) identified as men, 16 393 (54.1%) as women, and 193 (0.6%) as nonbinary. The most commonly endorsed cause was genetic factors (20 380 of 30 254; unweighted, 67.4%; weighted, 66.7%), an evidence-based risk factor. However, 8370 of 30 288 (unweighted, 27.6%; weighted, 26.6%) endorsed childhood vaccinations and 7140 of 30 211 (unweighted, 23.6%; weighted, 23.1%) endorsed acetaminophen in pregnancy as autism risk factors. In regression models, endorsement of vaccine-autism risk beliefs was greatest among adults aged 35 to 44 years (vs 18 to 24 years, adjusted odds ratio [aOR], 1.64; 95% CI, 1.44-1.86) and lowest among those aged 65 years and older (aOR, 0.64; 95% CI, 0.55-0.74). Endorsement was also associated with men (aOR, 1.26; 95% CI, 1.19-1.34), African American or Black race (compared with White race, aOR, 1.25; 95% CI, 1.15-1.35), Hispanic ethnicity (aOR, 1.13; 95% CI, 1.03-1.24), and strength of Republican political affiliation (strong Republican vs strong Democrat, aOR, 3.83; 95% CI, 3.47-4.24). More frequent social media use was associated with lower odds of endorsing misinformation (multiple times daily vs less than weekly, aOR, 0.72; 95% CI, 0.66-0.78).

**Conclusions and Relevance:**

Results of this survey study suggest that non–evidence-based beliefs about autism risk were prevalent among US adults and were strongly associated with sociodemographic differences and political affiliation. Limiting public health impact of such non–evidence-based information will likely require targeted interventions that recognize the heterogeneity of such beliefs.

## Key Points

**Question:**

What proportion of US adults endorse non–evidence-based beliefs about autism risk, and what characteristics are associated with these beliefs?

**Findings:**

In this cross-sectional survey study of 30 288 US adults, approximately one-quarter of respondents endorsed childhood vaccinations or acetaminophen use during pregnancy as autism risk factors. This endorsement was more common among individuals with particular sociodemographic characteristics and political affiliations, whereas greater trust in scientific and medical institutions was associated with lower rates of endorsement.

**Meaning:**

Results suggest that non–evidence-based beliefs about autism risk factors are common among US adults and highlight the need for targeted public health efforts to address these beliefs.

## Teaser

This survey study assesses associations between several sociodemographic characteristics and non–evidence-based beliefs about environmental autism risk factors through an internet-based survey of US adults.

## Introduction

Autism is among the most heritable common neurodevelopmental disorders, with an estimated 80% of autism risk explained by genetic variation.^1^ However, the observation that the apparent prevalence of autism and related disorders has increased over recent decades^2^ has led to greater focus on environmental risks, with a range of potential causes subject to speculation and, in some cases, investigation. Even if environmental risks account for a modest proportion of risk overall, the perception that such risk could be avoidable—or directly mitigated—has prompted abundant investment in considering these risks.

In 1998, a case series published in *Lancet* claimed to show that vaccination was associated with autism spectrum disorder (ASD) risk; 12 years later, the editors retracted this study^3^ after numerous flaws were identified. There has been no subsequent credible evidence supporting a link, whereas numerous studies have demonstrated no such association.^4,5^ Still, a number of surveys have suggested that vaccines remain linked to ASD in public perception. In a 2015 Gallup survey of US adults, although only 6% of adults believed vaccines were linked with autism, 52% were unsure; in 2019, similarly, 10% believed this link, whereas 46% were unsure.^6^ More recent surveys likewise indicate endorsement of this association. In a 2025 Kaiser Family Foundation poll, 23% of respondents said it was definitely or probably true that the measles-mumps-rubella vaccination had been proven to cause autism,^7^ whereas in a 2025 YouGov survey, 27% believed exposure to childhood vaccines contributes “a great deal” or “a fair amount” to autism risk.^8^

Another exposure suggested to increase ASD risk is maternal acetaminophen use,^9^ despite strong evidence against such an association.^10^ Similar to surveys on vaccination, a Kaiser Family Foundation survey in the fall of 2025 suggested uncertainty among US adults—although only 4% said it was definitely true that taking Tylenol during pregnancy increases autism risk, 30% said it was probably true. In that survey, Republicans were substantially more likely than Democrats to view the claim as true (56% vs 13%).^11^

Health-related misinformation is not a new phenomenon, but recent studies have suggested that social media has contributed to the generation and spread of such misinformation.^12,13,14^ Another key development has been the politicization of science, which predated the COVID-19 pandemic^15^ but was likely accelerated by differential messaging from political groups during the pandemic, with falling levels of trust in science and medicine itself.^16,17^ Although recent studies have established that a substantial minority of US adults endorses each of these risk claims, they were not designed to characterize who holds these beliefs or to examine the factors that may correlate with such beliefs. In this study, we therefore examined beliefs about autism risk factors to address 2 related goals. First, we sought to extend prior surveys with state-level estimates and characterize the extent to which US adults believe particular risk factors confer autism risk. Second, we aimed to understand what individual characteristics are associated with particular beliefs related to autism risk, including the role of political affiliation, social media use, and trust in institutions.

## Methods

### Study Design

This survey study was determined by the institutional review board of Harvard University to be exempt, as a low-risk study using deidentified data. All respondents provided electronic informed consent before answering survey questions. We used data from 1 wave (wave 37) of an internet survey conducted between December 18, 2025, and January 27, 2026, using the commercial service PureSpectrum. This survey marketplace aggregates participants from multiple opt-in, online, nonprobability research panels, rather than maintaining a single recruited panel. Panel members join by self-selection, typically in exchange for modest incentives which vary depending on the panel. As a nonprobability opt-in survey, response rate cannot be calculated. The survey is designed to facilitate state-level analyses,^18^ so 51 concurrent surveys are fielded—1 within each state and the District of Columbia—with state-level quotas for gender, age, and race and ethnicity. In addition to these state-level samples, the survey fields augmenting surveys targeting approximately 1000 completed responses each among Asian, Black, and Hispanic respondents. The survey incorporates open-ended questions aimed at confirming participant attention and minimizing automated or inattentive responses. The study was conducted in accordance with the American Association for Public Opinion Research (AAPOR) reporting guidelines (eMethods 1 in Supplement 1).^19^

### Measures

Knowledge of potential environmental risks for autism was examined by asking participants, “Is it true or false that the following factors can increase the risk of autism in children?” Possible answers were definitely true, probably true, probably false, definitely false, or not sure. The list of factors were chosen to range from those strongly supported by evidence to those strongly disproven, with inclusion of some for which evidence is mixed. Specific factors were genetic factors, childhood vaccinations, having an older father, being a boy, eating foods with artificial dyes, eating undercooked or raw fish, drinking water with fluoride, being born early in the pregnancy, mother using acetaminophen (Tylenol) during pregnancy, mother using antidepressants during pregnancy, mother having certain infections during pregnancy, and mother getting the tetanus, diphtheria, and pertussis vaccine during pregnancy. Among these, we considered genetic risk, older paternal age, male sex, premature birth, and maternal infection during pregnancy to be supported by evidence; antidepressant use during pregnancy to remain disputed; and the remainder to be non–evidence-based. We focused on acetaminophen and child vaccination, for which public figures have recently asserted a causal link despite the absence of supporting evidence. Per institutional review board policy for studies with potential misinformation, after this block of questions a summary of the evidence strength for individual items was provided to participants.

Sociodemographic features were drawn from participant self-report, spanning age, gender (man, woman, or multiple nonbinary terms), education, employment status, and race and ethnicity. The latter 2 could be selected from a checklist including African American or Black, Asian American, Hispanic, Native American, Pacific Islander, American Indian or Alaska Native, White, or other. Individuals checking other could provide a free text self-description. Race and ethnicity were collected in order to facilitate application of survey weights to ensure representativeness and are reported as advised in a recent guidance statement.^20^ To facilitate inclusion of smaller groups in models, we collapsed American Indian or Alaska Native, Native American, Pacific Islander, more than 1 race, and any other race not listed into a single category (termed *other*); categories are listed alphabetically (African American or Black, Asian American, other, and White), with Hispanic ethnicity reported separately.

Political affiliation was determined by first asking respondents their political party (Republican, Democrat, Independent, or other). Republicans and Democrats were then asked, “Do you consider yourself to be a strong/not very strong [Republican/Democrat]?” Independents and others were asked, “Do you think of yourself as closer to the Republican Party, the Democratic Party, or neither?” The answers yielded the 7 categories as follows: strong Republican, Republican, leaning Republican, Independent, leaning Democrat, Democrat, or strong Democrat. Strong indicates respondents who identified with a party and described themselves as a strong Republican or strong Democrat; Republican and Democrat are those who described themselves as not very strong; lean indicates respondents who identified as Independent or other but reported being closer to that party; and Independent indicates those closer to neither party.

Social media use by self-report was determined by asking, “Do you ever use any of the following social media sites or apps?” Respondents who answered affirmatively were then asked to indicate their frequency of overall use (less than once a week, once a week, several times a week, about once a day, several times a day, or most of the day). For analysis, these were collapsed to less than once a week, weekly to daily, and more than once a day.

Trust in individuals, professions, and institutions was assessed with, “How much do you trust the following people and organizations to do what is right?” followed by a specific group. The choices were a lot, some, not too much, and not at all. For primary analyses, we focused on hospitals and physicians (termed doctors in the survey) and scientists and researchers; we also examined trust in Donald Trump, colleges and universities, pharmaceutical companies, police, Centers for Disease Control and Prevention, and US Food and Drug Administration for comparison.

### Statistical Analysis

We report 2-sided *P* values with a statistical significance of *P* < .05. For descriptive purposes we did not apply weighting to generate the Table, but all subsequent results incorporate survey weights to approximate national distributions, with interlocking weights for age, gender, and race and ethnicity, for education and race and ethnicity, and for education and gender, together with US region and urbanicity. For state-level analyses, we applied the survey’s state-representative interlocking weights for poststratification raking to match each state’s composition by gender, race, age, education, and rurality. All weights relied on population estimates from the Census American Community Survey. For each state we report the unweighted number of respondents and the state-weighted proportion endorsing each belief, with 95% CIs computed on the logit scale. Unadjusted survey-weighted proportions endorsing each belief are reported for each characteristic. Consistent with prior reports from this survey, we additionally fit multivariable logistic regression models; because sociodemographic characteristics are correlated with one another, the unadjusted associations would not distinguish the contribution of any single characteristic from the others with which it covaries. Adjusted associations in linear and logistic regression models included age category, gender, education, annual household income, race and ethnicity, and rural, suburban, or urban setting.

We first characterized the proportion of individuals endorsing belief in each of the individual environmental risks. We then examined the proportions of individuals endorsing belief in the following 2 risk factors which, despite abundant evidence of nonassociation, remain subjects of public concern: acetaminophen exposure and vaccination. In regression models, we examined features associated with these beliefs. For comparison, we also examined the following established risk factors strongly supported by evidence: genetic effects, male sex, and prematurity. Further, we sought to understand the association with frequency of social media use and trust in institutions and individuals. To do so, we extended regression models with additional covariates for social media use and then for trust in specific groups. Lastly, in post hoc exploration to better understand the extent to which belief in risk factors was explained by a single factor, we conducted an exploratory principal components analysis of the 12 belief items. All analyses were conducted using R, version 4.4.2,^21^ and the R survey package,^22^ version 4.4-2 (R Foundation).

## Results

In all, 30 388 unique participants completed the survey. Of these, 100 (0.3%) did not provide a response to the childhood vaccination item and were excluded from analyses of that item, yielding an analytic sample of 30 288, with a mean age of 46.6 years (SD, 15.9 years). Among the participants, 16 393 (54.1%) identified as women, 13 702 (45.2%) as men, and 193 (0.6%) as nonbinary. In the analytic sample, 5449 (18.0%) identified as African American or Black, 1721 (5.7%) as Asian American, 21 066 (69.6%) as White, and 2052 (6.8%) as other; 4131 (13.6%) were Hispanic and 26 157 (86.4%) were non-Hispanic (eTable 1 in Supplement 1). Additional characteristics of the survey cohort are summarized in the Table.

**Table.** Baseline Characteristics of the Survey Cohort (Unweighted)

Characteristic	No. (%)		
	Overall (N = 30 288)^a^	Does not believe vaccines cause ASD (n = 21 918)	Believes vaccines cause ASD (n = 8370)
Age, mean (SD), y	46.6 (15.9)	47.6 (16.4)	43.8 (14.0)
Age group, y			
18-24	2268 (7.5)	1719 (7.8)	549 (6.6)
25-34	5471 (18.1)	3674 (16.8)	1797 (21.5)
35-44	6997 (23.1)	4636 (21.2)	2361 (28.2)
45-54	5774 (19.1)	4098 (18.7)	1676 (20.0)
55-64	4784 (15.8)	3594 (16.4)	1190 (14.2)
≥65	4994 (16.5)	4197 (19.1)	797 (9.5)
Gender			
Woman	16 393 (54.1)	12 276 (56.0)	4117 (49.2)
Man	13 702 (45.2)	9477 (43.2)	4225 (50.5)
Nonbinary	193 (0.6)	165 (0.8)	28 (0.3)
Race			
African American or Black	5449 (18.0)	3631 (16.6)	1818 (21.7)
Asian American	1721 (5.7)	1316 (6.0)	405 (4.8)
White	21 066 (69.6)	15 547 (70.9)	5519 (65.9)
Other^b^	2052 (6.8)	1424 (6.5)	628 (7.5)
Ethnicity			
Hispanic	4131 (13.6)	2807 (12.8)	1324 (15.8)
Not Hispanic	26 157 (86.4)	19 111 (87.2)	7046 (84.2)
Education			
Some high school or less	1319 (4.4)	920 (4.2)	399 (4.8)
High school graduate	8756 (28.9)	6123 (27.9)	2633 (31.5)
Some college	7385 (24.4)	5389 (24.6)	1996 (23.8)
College degree	9610 (31.7)	7178 (32.7)	2432 (29.1)
Graduate degree	3218 (10.6)	2308 (10.5)	910 (10.9)
Urbanicity			
Rural	5991 (19.8)	4307 (19.7)	1684 (20.1)
Suburban	16 172 (53.4)	11 937 (54.5)	4235 (50.6)
Urban	8125 (26.8)	5674 (25.9)	2451 (29.3)
Social media use frequency			
Less than weekly	5571 (18.4)	3746 (17.1)	1825 (21.8)
Weekly to daily	10 672 (35.2)	7575 (34.6)	3097 (37.0)
Multiple times daily	14 045 (46.4)	10 597 (48.3)	3448 (41.2)
Political affiliation^c^			
Strong Republican	5990 (19.9)	3462 (15.9)	2528 (30.3)
Republican	3563 (11.8)	2465 (11.3)	1098 (13.2)
Lean Republican	2061 (6.8)	1316 (6.0)	745 (8.9)
Independent	6420 (21.3)	4786 (21.9)	1634 (19.6)
Lean Democrat	2365 (7.8)	1976 (9.1)	389 (4.7)
Democrat	3529 (11.7)	2828 (13.0)	701 (8.4)
Strong Democrat	6235 (20.7)	4995 (22.9)	1240 (14.9)
Household income, $			
<25 000	7441 (24.6)	5372 (24.5)	2069 (24.7)
25 000-<50 000	7687 (25.4)	5623 (25.7)	2064 (24.7)
50 000-<75 000	5802 (19.2)	4247 (19.4)	1555 (18.6)
75 000-<100 000	3700 (12.2)	2680 (12.2)	1020 (12.2)
≥100 000	5658 (18.7)	3996 (18.2)	1662 (19.9)

Abbreviation: ASD, autism spectrum disorder.

^a^ Values are shown for the 30 288 respondents who provided a response to the childhood vaccination item; 100 of the 30 388 survey respondents did not and are not included.

^b^ The other race category comprises respondents identifying as American Indian or Alaska Native, multiracial, Native American, Pacific Islander, or another race not listed.

^c^ Strong indicates respondents who identified with a party and described themselves as a strong Republican or strong Democrat; Republican and Democrat are those who described themselves as not very strong; lean indicates respondents who identified as independent or other but reported being closer to that party; independent indicates those closer to neither party.

Figure 1 summarizes the survey-weighted proportion endorsing belief in each possible risk factor for autism, along with strength of belief, with eTable 1 in Supplement 1 reporting unweighted proportions. The most endorsed autism risk factor was genetic risk (20 380 of 30 254; unweighted, 67.4%; weighted, 66.7%; 95% CI, 66.1%-67.3%) and the least endorsed was male sex (5134 of 30 247; unweighted, 17.0%; weighted, 16.5% (95% CI, 16.0%-17.0%); 8370 of 30 288 endorsed childhood vaccinations (unweighted, 27.6%; weighted, 26.6%; 95% CI, 26.0%-27.1%) and 7140 of 30 211 endorsed acetaminophen during pregnancy (unweighted, 23.6%; weighted, 23.1%; 95% CI, 22.5%-23.6%).

**Figure 1.**
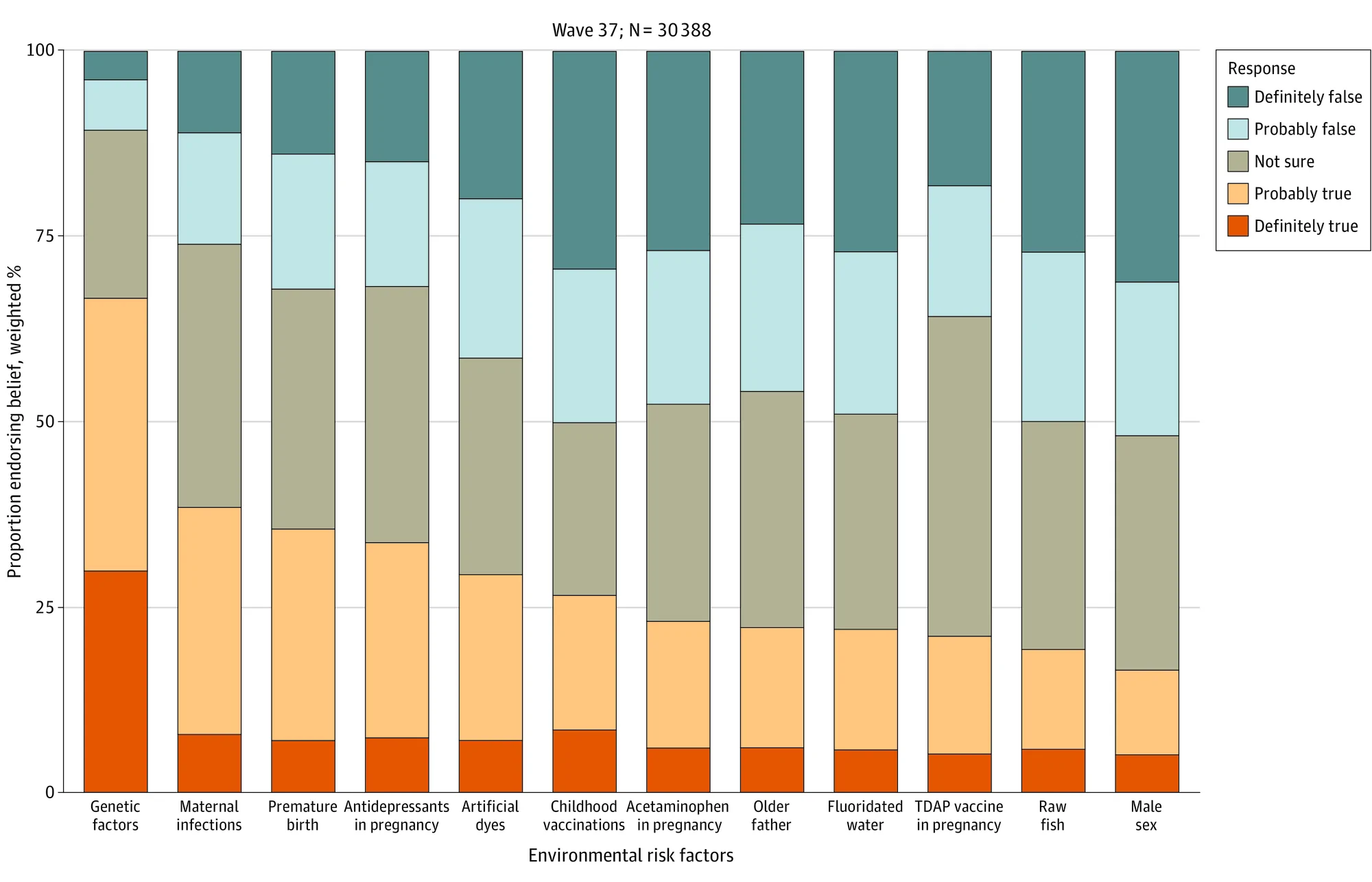
Bar Graph Showing Survey-Weighted Strength of Endorsement of Possible Autism Risk Factors Across All Items TDAP indicates tetanus, diphtheria, and pertussis.

Figure 2 illustrates the state-weighted proportion endorsing belief in childhood vaccination as a contributor to risk by state. Belief that vaccination is a risk factor varied modestly across states (median, 26.3%; IQR, 24.8%-27.9%), and it was highest in Alaska (33.8%; 95% CI, 28.3%-39.9%), North Dakota (32.7%; 95% CI, 26.8%-39.3%), and Florida (31.2%; 95% CI, 28.4%-34.2%) and lowest in Vermont (20.6%; 95% CI, 15.8%-26.4%), Hawaii (22.1%; 95% CI, 17.4%-27.6%), and Wisconsin (22.3%; 95% CI, 18.7%-26.4%). Corresponding results for acetaminophen (and, as a positive control, genetic risk) are presented in eFigures 1 and 2 and eTable 3 in Supplement 1.

**Figure 2.**
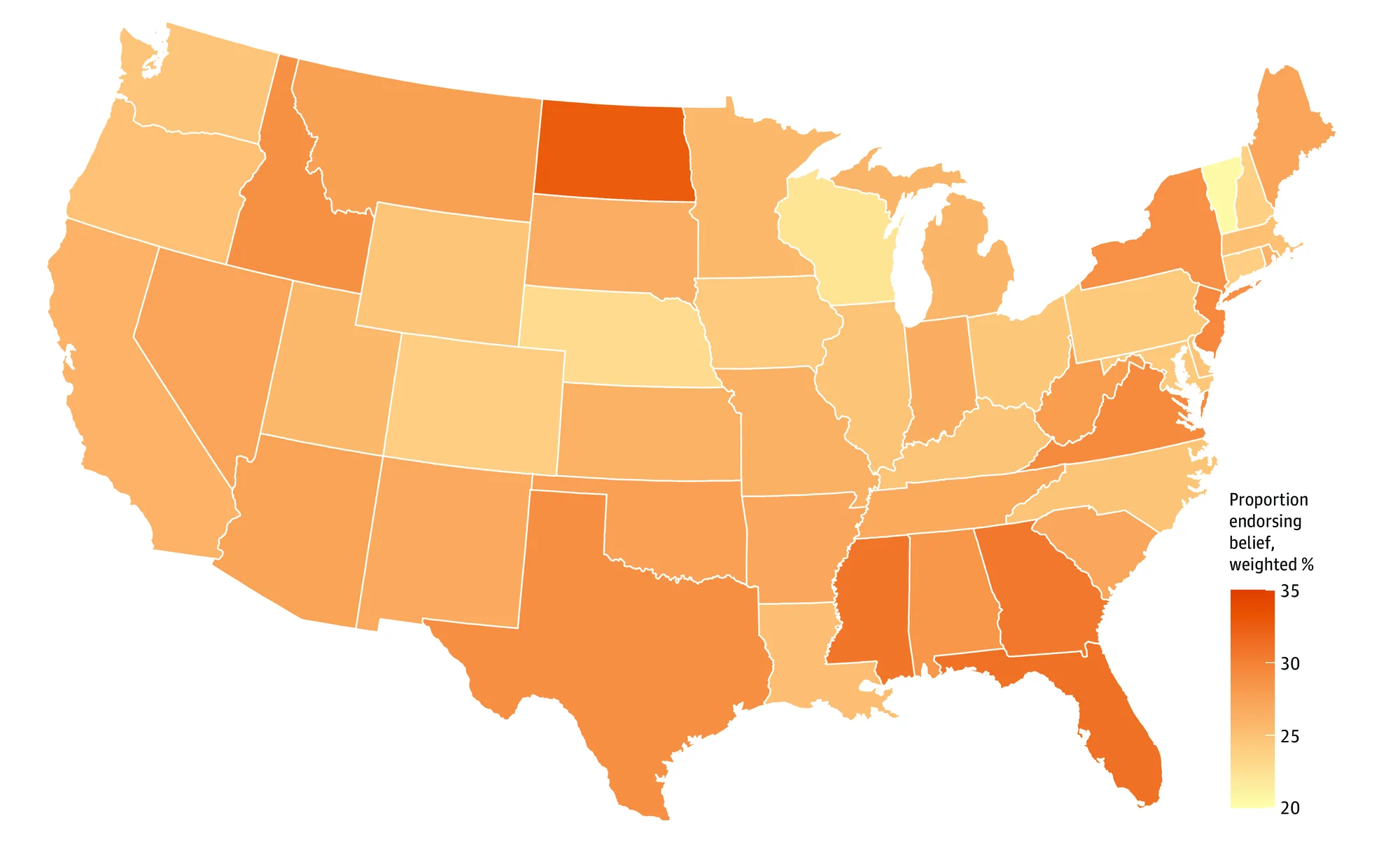
Map of State-Weighted Endorsement of Childhood Vaccination as an Autism Risk Factor, by US State State-weighted proportion endorsing probably or definitely true; all 50 states and the District of Columbia included. State-representative survey weights were applied; per-state sample sizes and 95% CIs are provided in eTable 3 in Supplement 1. Alaska, Hawaii, and District of Columbia were tabulated but are not shown on base map.

We next examined sociodemographic and other characteristics associated with autism risk factor beliefs, using multivariable logistic regression models in the full national dataset and adjusting for all sociodemographic covariates (Figure 3; both unadjusted and adjusted odds ratios [aOR] for each characteristic are reported in eTable 2 in Supplement 1). Belief in childhood vaccination as a risk factor followed an inverted-U pattern across age—compared with adults aged 18 to 24 years, odds were higher among those aged 25 to 34 years (aOR, 1.58; 95% CI, 1.39-1.80), 35 to 44 years (aOR, 1.64; 95% CI, 1.44-1.86), and 45 to 54 years (aOR, 1.37; 95% CI, 1.20-1.56), but were not significantly different at 55 to 64 years (aOR, 1.11; 95% CI, 0.96-1.27) and were lowest among those aged 65 years and older (aOR, 0.64; 95% CI, 0.55-0.74). Endorsement of risk was more likely among individuals who were men (aOR, 1.26; 95% CI, 1.19-1.34) and of African American or Black race (compared with White race, aOR, 1.25; 95% CI, 1.15-1.35). Hispanic ethnicity was also associated with greater likelihood of endorsing this risk (aOR, 1.13; 95% CI, 1.03-1.24). Adding political party to this model, all other affiliations were less likely to endorse this belief than strong Republican (aORs, 0.23-0.71; strong Republican vs strong Democrat, aOR, 3.83; 95% CI, 3.47-4.24; eFigure 3 in Supplement 1).

**Figure 3.**
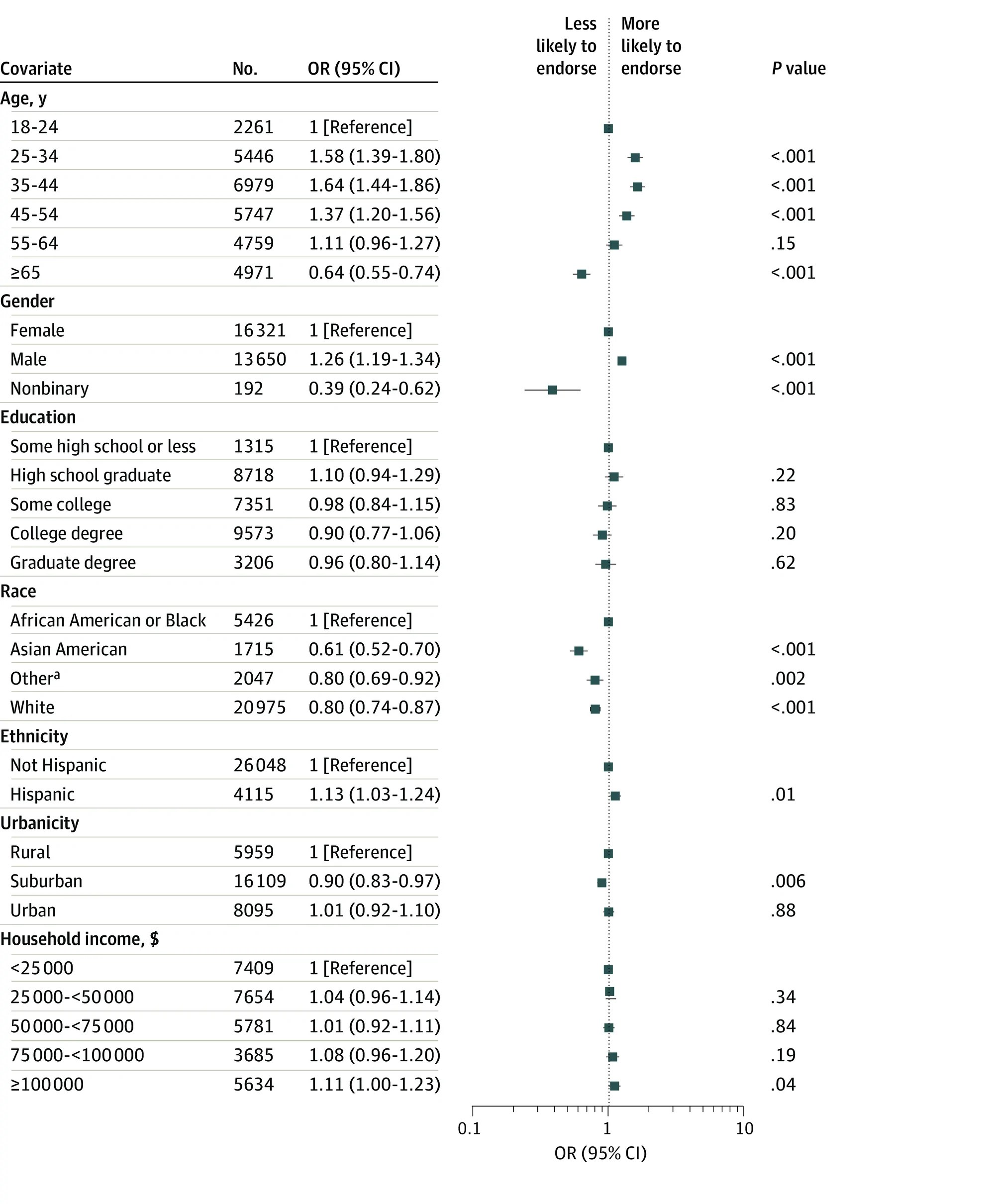
Forest Plot of Multivariable Logistic Regression for Endorsing Childhood Vaccination as an Autism Risk Factor OR indicates odds ratio. ^a^The other race category comprises respondents identifying as American Indian or Alaska Native, multiracial, Native American, Pacific Islander, or another race not listed.

For acetaminophen (eFigure 4 in Supplement 1), associated features similarly included men (aOR, 1.39; 95% CI, 1.30-1.48) and African American or Black race (compared with White race, aOR, 1.26; 95% CI, 1.16-1.37). Here age effects differed, with older age associated with lower belief (ages ≥65 years vs 18-24 years, aOR, 0.43; 95% CI, 0.37-0.50). As with vaccination, all political affiliations other than strong Republican (eFigure 5 in Supplement 1) were less likely to endorse this belief (aORs, 0.26-0.67).

We next examined the association between the use of social media and belief in vaccination as a risk factor. Here results showed an inverse gradient (eFigure 6 in Supplement 1): more frequent social media use was associated with lower likelihood of belief in vaccination as a risk factor—compared with less than weekly use, those using social media weekly to daily had lower odds of vaccine belief (aOR, 0.87; 95% CI, 0.80-0.95), as did those using social media multiple times daily (aOR, 0.72; 95% CI, 0.66-0.78). A similar pattern held for acetaminophen as a risk factor (eFigure 7 in Supplement 1)—those using social media multiple times daily had lower odds of acetaminophen belief (aOR, 0.79; 95% CI, 0.72-0.86).

We also examined the association between trust in institutions and endorsement of non–evidence-based risk factors. For both vaccination (Figure 4) and acetaminophen use (eFigure 8 in Supplement 1), greater trust in scientists and clinicians was associated with a lesser likelihood of endorsing these beliefs—for a lot vs not at all trust, aORs for vaccination were 0.34 (95% CI, 0.29-0.40) for scientists and researchers and 0.47 (95% CI, 0.40-0.56) for hospitals and physicians; corresponding aORs for acetaminophen were 0.49 (95% CI, 0.42-0.58) and 0.64 (95% CI, 0.53-0.76). Trust in Donald Trump was associated with a significantly greater likelihood of such beliefs (vaccination aOR, 4.51 [95% CI, 4.13-4.91]; acetaminophen aOR, 4.50 [95% CI, 4.11-4.93]). For genetic risk (eFigure 9 in Supplement 1), the pattern reversed. Greater trust in scientists and researchers (aOR, 2.58; 95% CI, 2.23-3.00) and in hospitals and physicians (aOR, 2.30; 95% CI, 1.95-2.71) was associated with greater likelihood of belief. Trust in Donald Trump was associated with lesser likelihood of endorsing this belief (aOR, 0.90; 95% CI, 0.83-0.98).

**Figure 4.**
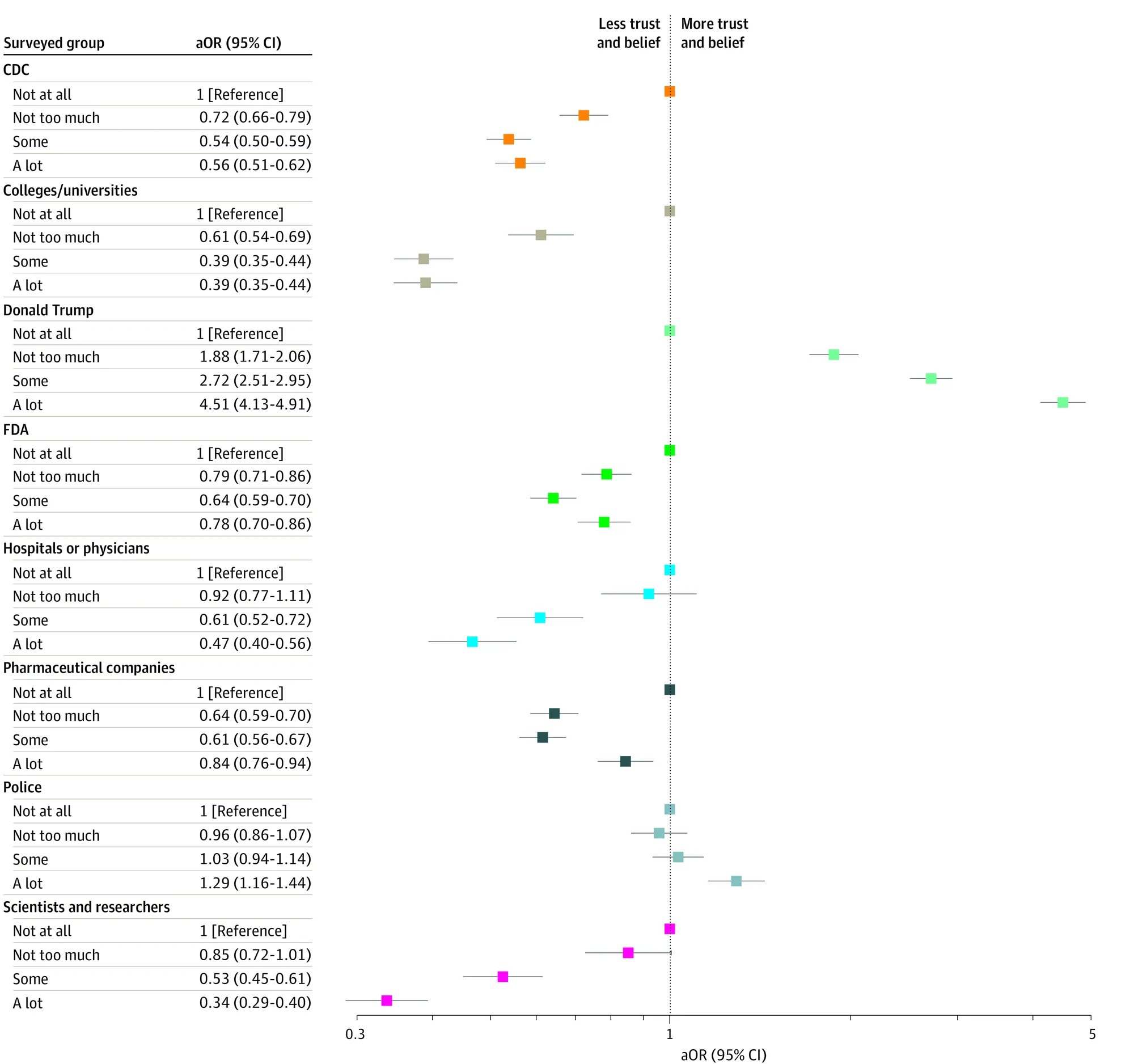
Forest Plot of Results From Multivariable Logistic Regression Models of Trust in Institutions or Individuals and Endorsement of Childhood Vaccination as an Autism Risk Factor, Adjusted for Sociodemographic Features aOR indicates adjusted odds ratio; CDC, Centers for Disease Control and Prevention; FDA, US Food and Drug Administration.

We then conducted an exploratory principal components analysis of the 12 belief items (eMethods 2, eFigure 10, and eTable 4 in Supplement 1). This analysis retained 3 promax-rotated components accounting for 67.4% of the variance—a general component loading on the non–evidence-based and disputed causes (general component, 40.7%), consistent with a general tendency to endorse contested claims; a component loading on the evidence-based biological causes (evidence-based component, 11.8%; genetic factors, prematurity, and maternal infection); and a component loading on demographic characteristics (demographic component, 14.9%; older paternal age and male sex). The general and evidence-based components were only modestly correlated (survey-weighted correlation of component scores, *r* = 0.24; 95% CI, 0.22 to 0.25). Greater trust in physicians and scientists (a lot vs not at all) was associated with lower scores on the general nonevidence component (adjusted difference in component score, scientists and researchers, −0.63 [95% CI, −0.70 to −0.55]; hospitals and physicians, −0.46 [95% CI, −0.55 to −0.37]) but higher scores on the evidence-based component (scientists and researchers, 0.35 [95% CI, 0.27 to 0.44]; hospitals and physicians, 0.31 [95% CI, 0.22 to 0.41]) (eTable 5 in Supplement 1). These results suggest that endorsement of misinformation does not simply reflect a general propensity to accept any proposed cause (eg, acquiescence bias), but a distinct orientation aligned with distrust of medical and scientific institutions.

## Discussion

Among 30 288 adults from all 50 US states and the District of Columbia participating in an internet survey, we found that non–evidence-based beliefs about risks for autism were prevalent. They exhibited marked sociodemographic and modest geographic variability but consistent association with political affiliation—greater Republican affiliation was associated with greater likelihood of endorsing non–evidence-based beliefs about risk.

Our results add to abundant evidence of the growing politicization of beliefs about science and medicine. This politicization of science preceded the COVID-19 pandemic,^15^ but variability in responses to the pandemic by state and federal leadership likely accelerated this trend. Prior survey results document a decline in trust in health care^16^ and science^15,17^ during the pandemic. Our results may reflect this diminished trust in traditional sources of public health information. In fact, we find that trust in these sources of medical and scientific facts is inversely related to belief in non–evidence-based risk.

Conversely, although social media use has been blamed for accelerating dissemination of misinformation,^14,23^ we did not identify a positive association between social media use and belief in non–evidence-based risk factors. Indeed, more frequent use was associated with a lower likelihood of endorsing such beliefs. One possibility is that social media use parallels information seeking more generally—ie, those with lower levels of use are also those with less access to sources of reliable health information.

In aggregate, our results suggest the complexity of public health messaging in the post–COVID-19 era. The large proportion of the population holding beliefs that may adversely impact public health (eg, by preventing vaccination) highlights the magnitude of the problem. The extent to which these beliefs are associated with particular population subgroups also underscores the need for more targeted interventions, rather than using single broad channels to disseminate medical and scientific news.

### Limitations

This study has some limitations. First, in light of its cross-sectional design, we can only identify associations and cannot establish causation (for example, an association between beliefs and moderate-intensity social media use does not confirm that such use prevents or contributes to those beliefs). We capture social media use by self-report, which may not yield reliable estimates of actual use.^24^ Furthermore, the study’s nonprobability design precludes calculating a response rate and excluding biases arising from differential self-selection into the survey. Some methodological studies identify challenges in online surveys, probability or otherwise, when they are not carefully designed. Our survey applied fraud and duplication screening as well as open-ended attention-check items reviewed for nonsubstantive or automated content (eMethods 1 in Supplement 1). Of course, no design can fully exclude fraudulent responses; this is true even for probability surveys using internet and phone-based responses.

We recognize that nonprobability surveys have been criticized as less accurate than traditional probability designs.^25,26,27,28^ Some of these comparisons, however, rely on data from periods when response rates in probability samples were notably higher than they are today (in telephone surveys, 36% in 1997 vs 6% in 2018),^26,27,29,30^ with growing nonignorable nonresponse,^31^ and many ignore the reality that nonprobability samples vary widely in quality.^32,33^ Nonprobability sample surveys can perform as well as—or sometimes better than—probability sample surveys; for example, matching administrative benchmarks for COVID-19 vaccination before booster rollout^34^ and infection rates,^35^ performing comparably to telephone probability polls in the 2020 presidential election^36,37,38^ and predicting presidential vote choice in 2024,^39^ and characterizing patterns of firearm ownership consistent with external benchmarks.^40^ In our results, overall endorsement of childhood vaccination as an autism risk factor (26.6%) is close to the 27% reported by a fall 2025 YouGov high-quality nonprobability survey^8^ and to the 23% reported by a 2025 Kaiser Family Foundation probability poll,^7^ neither of which was powered for subgroup estimates.

## Conclusions

Results of this survey study suggest that a large subset of US adults held beliefs about autism risk factors that have been repeatedly debunked, and that these beliefs were associated with political affiliation. In light of the potential public health impact of diminished childhood vaccinations, these results may be valuable in informing efforts to improve health literacy among groups at particularly high risk for having such beliefs.

## Notes

Supplement 1. **eMethods 1.** AAPOR Survey Disclosure Checklist and Additional Methodological Detail **eTable 1. **Unadjusted Survey-Weighted Proportion Endorsing Childhood Vaccination and Acetaminophen, Within Each Characteristic **eTable 2. **Unadjusted and Adjusted Survey-Weighted Odds Ratios for Endorsing Childhood Vaccination and Acetaminophen **eFigure 1. **State-Weighted Endorsement of Acetaminophen in Pregnancy as an Autism Risk Factor, By State **eFigure 2. **State-Weighted Endorsement of Genetic Factors as an Autism Risk Factor, By State **eTable 3. **Per-State Unweighted Sample Size and State-Weighted Proportion Endorsing Each Belief **eFigure 3. **Multivariable Model for Childhood Vaccination, Adding Political Affiliation **eFigure 4. **Multivariable Model for Acetaminophen in Pregnancy **eFigure 5. **Multivariable Model for Acetaminophen, Adding Political Affiliation **eFigure 6. **Social Media Use and Endorsement of Childhood Vaccination **eFigure 7. **Social Media Use and Endorsement of Acetaminophen **eFigure 8. **Trust in Institutions and Endorsement of Acetaminophen **eFigure 9. **Trust in Institutions and Endorsement of Genetic Factors **eMethods 2. **Principal Components Analysis of the 12 Belief Items **eFigure 10. **Scree Plot and Parallel Analysis for the Principal Components Analysis **eTable 4. **Survey-Weighted Promax-Rotated Principal Component Loadings **eTable 5. **Association of Trust (a Lot vs Not At All) With Each Component Score

Supplement 2.
Data Sharing Statement.
