# Supplementary material for: Knowledge and Misinformation Regarding Autism Risk Factors in a Survey of US Adults
Source: JAMA Health Forum. 2026 Nov 6;7(11):e264105. doi: 10.1001/jamahealthforum.2026.4105 (PMC13744024; doi:10.1001/jamahealthforum.2026.4105)
Supplement: Supplementary file 1 [file jamahealthforum-e264105-s001.pdf]

Supplementary Online Content

Perlis RH, Santillana M, Baum MA, Druckman JN, Ognyanova K, Lazer D. Knowledge and misinformation regarding autism risk factors in a survey of US adults. *JAMA Health Forum*. Published online November 6, 2026. doi:10.1001/jamahealthforum.2026.4105

eMethods 1. AAPOR Survey Disclosure Checklist and Additional Methodological Detail

eTable 1. Unadjusted Survey-Weighted Proportion Endorsing Childhood Vaccination and Acetaminophen, Within Each Characteristic

eTable 2. Unadjusted and Adjusted Survey-Weighted Odds Ratios for Endorsing Childhood Vaccination and Acetaminophen

eFigure 1. State-Weighted Endorsement of Acetaminophen in Pregnancy as an Autism Risk Factor, By State

eFigure 2. State-Weighted Endorsement of Genetic Factors as an Autism Risk Factor, By State

eTable 3. Per-State Unweighted Sample Size and State-Weighted Proportion Endorsing Each Belief

eFigure 3. Multivariable Model for Childhood Vaccination, Adding Political Affiliation

eFigure 4. Multivariable Model for Acetaminophen in Pregnancy

eFigure 5. Multivariable Model for Acetaminophen, Adding Political Affiliation

eFigure 6. Social Media Use and Endorsement of Childhood Vaccination

eFigure 7. Social Media Use and Endorsement of Acetaminophen

eFigure 8. Trust in Institutions and Endorsement of Acetaminophen

eFigure 9. Trust in Institutions and Endorsement of Genetic Factors

eMethods 2. Principal Components Analysis of the 12 Belief Items

eFigure 10. Scree Plot and Parallel Analysis for the Principal Components Analysis

eTable 4. Survey-Weighted Promax-Rotated Principal Component Loadings

© 2026 Perlis RH et al. *JAMA Health Forum*.

eTable 5. Association of Trust (a Lot vs Not At All) With Each Component Score

This supplementary material has been provided by the authors to give readers additional information about their work.

© 2026 Perlis RH et al. *JAMA Health Forum*.

eMethods 1. AAPOR Survey Disclosure Checklist and additional methodological detail

1. Data collection strategy. Non-probability, quota-sampled online survey administered as wave 37 of an ongoing multi-wave research program (the COVID States Project / Civic Health and Institutions Project, CHIP-50). Implemented as 51 individual surveys (one per state, plus District of Columbia), each with its own quotas.

2. Sponsor and conductor. Funders and the academic institutions conducting the work are named in the Acknowledgment. Survey was executed by PureSpectrum.

3. Measurement tools. Verbatim wording, response options, and question order for the autism risk-factor battery, the social media use items, and the trust battery are reproduced below under 'Survey items'.

4. Population under study. Adults aged 18 years or older residing in the 50 US states and the District of Columbia.

5. Sample generation and recruitment. PureSpectrum is a commercial survey aggregator that aggregates respondents from multiple opt-in online panels rather than a single panel. Respondents are not recruited from a probability-based frame; panel composition varies over time and participants opt in, such that no sampling frame or coverage rate can be specified. Interlocking quotas (gender, age, race and ethnicity) were applied within each state and DC, set to state population margins based on US Census American Community Survey data. In addition to these state-level samples, the survey fields augments targeting approximately 1,000 completed responses each among Black, Hispanic, and Asian respondents.

6. Method and mode of data collection. Self-administered web survey, desktop and mobile.

7. Dates of data collection. December 18, 2025 to January 27, 2026.

8. Sample size and precision. N = 30,388 completed responses; 30,288 of these provided a response to the childhood vaccination item that serves as the primary outcome. As a non-probability sample, a margin of sampling error cannot be validly calculated.

9. Weighting. Post-stratification raking to Census American Community Survey margins with interlocking weights for age, gender, and race and ethnicity; for education and race and ethnicity; and for education and gender; together with US region and urbanicity. National and state weights were trimmed at the 1st and 99th percentiles.

10. Data processing and data quality. The CHIP50 data include a set of quality control criteria, applied during processing and before any analysis.

Quality control criteria, part 1: removal criteria. Respondents are removed outright if any of the following hold:

(1) they fail either of two attention checks that ask them to select a specific response option;

© 2026 Perlis RH et al. *JAMA Health Forum*.

(2) they decline the survey pledge to provide their best answers;

(3) they do not complete the survey;

(4) they arrive with a missing or test panel ID;

(5) their total survey duration is 300 seconds or less;

(6) they indicate they were recruited through Amazon Mechanical Turk;

(7) any quota demographic (age, gender, race, education, or state) remains missing after all available sources are reconciled;

(8) they report residence outside the 50 states and the District of Columbia;

(9) they submit a second completed response.

Quality control criteria, part 2: quality checks. The remaining respondents are evaluated on a series of quality checks:

(1) prevalence of item non-response, flagged at more than 30 unanswered items or an unanswered percentage above 5%;

(2) straight-lining of consecutive item responses, flagged at a straightlining percentage above 0.5;

(3) short survey completion time, flagged in the fastest 5% of valid responses;

(4) saying yes to an impossible claim item asking whether the respondent is licensed to operate a class SSGN submarine;

(5) Google's invisible reCAPTCHA v3, which returns a score on which low values indicate a likely bot, flagged below 0.5;

(6) RelevantID fraud detection, flagged at a score of 30 or above, with a missing score also treated as flagged.

Respondents who fail two or more of these checks are removed. Failing a single check does not result in removal, and the count of checks failed is retained for the respondents who remain.

11. Statement of limitations. Because PureSpectrum aggregates supply from multiple panels whose identities and relative contributions are proprietary to the vendor and vary over the field period, the constituent panels cannot be enumerated and a cumulative response rate cannot be computed. See the manuscript Limitations.

Assessment of missing data. Responses were not forced, and respondents could advance without answering an item; item-level missingness for the twelve belief items was nonetheless low, ranging from 50 respondents (0.16%) for genetic factors to 164 (0.54%) for maternal TDaP vaccination. 'Not sure' was an explicit response option and is not treated as missing. Among model covariates, only political

© 2026 Perlis RH et al. *JAMA Health Forum*.

affiliation exhibited missingness (127 respondents, 0.42%); age, gender, education, income, race, ethnicity, urbanicity, and social media use had none. There is no follow-up in this cross-sectional design, so no participants were lost to follow-up. Of the 30,388 respondents, 100 (0.3%) did not provide a response to the childhood vaccination item and are excluded from analyses of that item, yielding the analytic sample of 30,288; 29,604 respondents provided a response to all twelve belief items and contribute to the principal components analysis.

Survey items

Autism risk factors. Question stem: 'Is it true or false that the following factors can increase the risk of autism in children?'

Items, in the order presented: Genetic factors; Childhood vaccinations; Having an older father; Being a boy; Eating foods with artificial dyes; Eating undercooked or raw fish; Drinking water with fluoride; Being born early in the pregnancy; Mother using Tylenol during pregnancy; Mother using antidepressants during pregnancy; Mother having certain infections during pregnancy; Mother getting the TDaP vaccine during pregnancy.

Response options, in the order presented: Definitely true; Probably true; Probably false; Definitely false; Not sure. Responses are stored in the source data as Definitely false = 1, Probably false = 2, Probably true = 3, Definitely true = 4, and Not sure = 0. For the principal components analysis these were recoded to a 5-point ordinal scale (Definitely false = 1, Probably false = 2, Not sure = 3, Probably true = 4, Definitely true = 5); the sensitivity analysis treated Not sure as missing, giving a 4-point scale. Per institutional review board policy for studies presenting potential misinformation, a summary of the strength of evidence for each item was provided to participants after this block.

Social media use. Question stem: 'Do you ever use any of the following social media sites or apps?' (Please select all that apply).

Platforms listed, in the order presented: BeReal; Bluesky; Gab; Facebook; Facebook Messenger; Instagram; Lemon8; LinkedIn; Mastodon; Parler; Pinterest; Post; Reddit; RedNote; Truth Social; TikTok; Tumblr; X (Twitter); Twitch; Snapchat; YouTube; WhatsApp; 4chan.

Respondents selecting one or more platforms were then asked, for each platform selected: 'How often do you use the following social media platforms?' Response options, in the order presented: Most of the day; Several times a day; About once a day; Several times a week; Once a week; Less than once a week. For analysis, the highest frequency reported across any platform was used, and collapsed to three categories: less than once a week (including respondents reporting no platform use), weekly to daily, and more than once a day.

Trust. Question stem, presented separately for each entity: 'How much do you trust the following people and organizations to do what is right?'

© 2026 Perlis RH et al. *JAMA Health Forum*.

Entities analyzed in this report: The FDA (Food and Drug Administration); The CDC (Centers for Disease Control and Prevention); Donald Trump; Hospitals and doctors; Pharmaceutical companies; Scientists and researchers; Colleges and universities; The police.

Response options, in the order presented: A lot = 4; Some = 3; Not too much = 2; Not at all = 1.

AAPOR = American Association for Public Opinion Research.

© 2026 Perlis RH et al. *JAMA Health Forum*.

eTable 1. Unadjusted survey-weighted proportion endorsing childhood vaccination and acetaminophen use during pregnancy as autism risk factors, within each level of each characteristic

Characteristic	Childhood vaccination, No.	Childhood vaccination, weighted % (95% CI)	Acetaminophen in pregnancy, No.	Acetaminophen in pregnancy, weighted % (95% CI)
**Age group, years**				
18 to 24	2268	23.6 (21.6-25.7)	2268	26.8 (24.7-28.9)
25 to 34	5471	32.7 (31.3-34.1)	5469	30.4 (29.0-31.8)
35 to 44	6997	33.5 (32.3-34.7)	7007	28.9 (27.8-30.1)
45 to 54	5774	29.7 (28.3-31.1)	5784	22.7 (21.4-23.9)
55 to 64	4784	25.1 (23.7-26.5)	4775	18.8 (17.6-20.1)
65 and over	4994	16.0 (14.8-17.2)	4990	13.5 (12.5-14.7)
**Gender**				
Female	16393	24.1 (23.3-24.8)	16400	19.9 (19.3-20.6)
Male	13702	29.5 (28.6-30.4)	13699	26.6 (25.7-27.5)
Nonbinary	193	11.3 (7.3-17.0)	194	8.2 (4.9-13.6)
**Education**				
Some High School or Less	1319	27.3 (24.6-30.2)	1319	24.7 (22.0-27.5)
High School Graduate	8756	28.0 (26.9-29.1)	8758	24.1 (23.1-25.2)
Some College	7385	26.0 (24.9-27.1)	7389	20.9 (19.9-22.0)
College Degree	9610	24.8 (23.9-25.7)	9610	22.1 (21.2-23.0)
Graduate Degree	3218	26.8 (25.2-28.4)	3217	26.4 (24.8-28.0)
**Household income**				
<$25K	7441	26.5 (25.4-27.7)	7442	23.4 (22.3-24.6)
$25K-<$50K	7687	26.1 (24.9-27.2)	7690	21.4 (20.4-22.5)
$50K-<$75K	5802	25.6 (24.4-27.0)	5799	21.1 (19.9-22.3)
$75K-<$100K	3700	27.0 (25.4-28.7)	3702	23.8 (22.3-25.5)
$100K+	5658	28.0 (26.7-29.3)	5660	26.5 (25.2-27.8)
**Race**				
African American or Black	5449	32.0 (30.6-33.5)	5452	28.6 (27.2-30.0)
Asian American	1721	22.0 (19.9-24.3)	1721	19.4 (17.3-21.5)
Other	2052	29.8 (27.4-32.2)	2057	25.7 (23.5-28.1)
White	21066	25.5 (24.8-26.2)	21063	21.9 (21.3-22.6)
**Ethnicity**				
Not Hispanic	26157	25.6 (25.0-26.2)	26161	22.1 (21.5-22.7)

© 2026 Perlis RH et al. *JAMA Health Forum*.

Hispanic	4131	31.4 (29.7-33.1)	4132	28.0 (26.4-29.7)
**Urbanicity**				
Rural	5991	26.9 (25.6-28.1)	5997	22.9 (21.7-24.2)
Suburban	16172	25.1 (24.4-25.9)	16174	21.3 (20.5-22.0)
Urban	8125	29.0 (27.9-30.2)	8122	26.4 (25.3-27.5)
**Social media use frequency**				
Less than weekly	5571	31.4 (30.0-32.9)	5569	26.1 (24.8-27.5)
Weekly to daily	10672	28.1 (27.1-29.0)	10686	24.9 (24.0-25.9)
Multiple times daily	14045	23.5 (22.7-24.3)	14038	20.4 (19.7-21.2)
**Political affiliation**				
Strong Republican	5990	41.5 (40.0-42.9)	5993	36.4 (35.0-37.8)
Republican	3563	30.1 (28.4-31.8)	3563	25.6 (23.9-27.3)
Lean Republican	2061	34.7 (32.3-37.1)	2056	28.1 (25.9-30.5)
Independent	6420	24.6 (23.4-25.8)	6425	20.1 (19.0-21.3)
Lean Democrat	2365	15.9 (14.2-17.6)	2368	14.6 (13.0-16.4)
Democrat	3529	18.4 (17.0-19.9)	3532	18.6 (17.2-20.2)
Strong Democrat	6235	18.5 (17.4-19.6)	6231	15.9 (14.9-17.0)

Proportions are survey-weighted to approximate national distributions (interlocking weights for age, gender, race and ethnicity, education, US region, and urbanicity); 95% CIs were computed on the logit scale. No. is the unweighted number of respondents within each level who provided a response to that item: 30,288 for childhood vaccination and 30,293 for acetaminophen. Counts for political affiliation are lower (30,163 and 30,168) because 125 respondents did not report political affiliation.

© 2026 Perlis RH et al. *JAMA Health Forum*.

eTable 2. Unadjusted and adjusted survey-weighted odds ratios for endorsing childhood vaccination and acetaminophen use during pregnancy as autism risk factors

Characteristic	Childhood vaccination Unadjusted OR (95% CI)	Childhood vaccination Adjusted OR (95% CI)	Acetaminophen in pregnancy Unadjusted OR (95% CI)	Acetaminophen in pregnancy Adjusted OR (95% CI)
**Age group, years**				
18 to 24 [Reference]	1 [Reference]	1 [Reference]	1 [Reference]	1 [Reference]
25 to 34	1.58 (1.39-1.80)	1.58 (1.39-1.80)	1.20 (1.06-1.36)	1.14 (1.00-1.30)
35 to 44	1.63 (1.44-1.84)	1.64 (1.44-1.86)	1.11 (0.99-1.26)	1.05 (0.93-1.19)
45 to 54	1.36 (1.20-1.55)	1.37 (1.20-1.56)	0.80 (0.71-0.91)	0.77 (0.67-0.87)
55 to 64	1.08 (0.95-1.24)	1.11 (0.96-1.27)	0.64 (0.56-0.73)	0.62 (0.54-0.72)
65 and over	0.62 (0.53-0.71)	0.64 (0.55-0.74)	0.43 (0.37-0.50)	0.43 (0.37-0.50)
**Gender**				
Female [Reference]	1 [Reference]	1 [Reference]	1 [Reference]	1 [Reference]
Male	1.32 (1.24-1.40)	1.26 (1.19-1.34)	1.45 (1.37-1.55)	1.39 (1.30-1.48)
Nonbinary	0.40 (0.25-0.64)	0.39 (0.24-0.62)	0.36 (0.21-0.63)	0.30 (0.17-0.52)
**Education**				
Some High School or Less [Reference]	1 [Reference]	1 [Reference]	1 [Reference]	1 [Reference]
High School Graduate	1.04 (0.89-1.21)	1.10 (0.94-1.29)	0.97 (0.83-1.14)	1.05 (0.89-1.24)
Some College	0.94 (0.81-1.09)	0.98 (0.84-1.15)	0.81 (0.69-0.95)	0.87 (0.74-1.03)
College Degree	0.88 (0.76-1.02)	0.90 (0.77-1.06)	0.87 (0.74-1.02)	0.94 (0.80-1.12)
Graduate Degree	0.98 (0.83-1.15)	0.96 (0.80-1.14)	1.09 (0.92-1.30)	1.13 (0.93-1.36)
**Household income**				
<$25K [Reference]	1 [Reference]	1 [Reference]	1 [Reference]	1 [Reference]
$25K-<$50K	0.98 (0.90-1.06)	1.04 (0.96-1.14)	0.89 (0.82-0.98)	0.96 (0.87-1.05)
$50K-<$75K	0.95 (0.87-1.05)	1.01 (0.92-1.11)	0.87 (0.79-0.96)	0.90 (0.82-1.00)
$75K-<$100K	1.03 (0.93-1.14)	1.08 (0.96-1.20)	1.02 (0.92-1.14)	1.03 (0.92-1.15)
$100K+	1.08 (0.99-1.18)	1.11 (1.00-1.23)	1.18 (1.08-1.29)	1.13 (1.01-1.26)
**Race**				
White [Reference]	1 [Reference]	1 [Reference]	1 [Reference]	1 [Reference]
African American or Black	1.37 (1.27-1.48)	1.25 (1.15-1.35)	1.42 (1.32-1.54)	1.26 (1.16-1.37)
Asian American	0.82 (0.72-0.94)	0.76 (0.66-0.87)	0.86 (0.74-0.98)	0.73 (0.63-0.84)
Other	1.23 (1.09-1.39)	1.00 (0.87-1.14)	1.23 (1.09-1.40)	0.96 (0.83-1.10)
**Ethnicity**				
Not Hispanic [Reference]	1 [Reference]	1 [Reference]	1 [Reference]	1 [Reference]
Hispanic	1.32 (1.21-1.44)	1.13 (1.03-1.24)	1.37 (1.25-1.49)	1.16 (1.05-1.28)

© 2026 Perlis RH et al. *JAMA Health Forum*.

**Urbanicity**				
Rural [Reference]	1 [Reference]	1 [Reference]	1 [Reference]	1 [Reference]
Suburban	0.92 (0.85-0.99)	0.90 (0.83-0.97)	0.91 (0.84-0.99)	0.86 (0.79-0.94)
Urban	1.12 (1.03-1.22)	1.01 (0.92-1.10)	1.21 (1.10-1.32)	1.03 (0.93-1.13)

Odds ratios are survey-weighted to approximate national distributions. Unadjusted ORs are from single-predictor logistic models; adjusted ORs are from the multivariable models reported in the main text. Both models adjust for age, gender, education, household income, race, ethnicity, and urbanicity; these are the Model 1 specifications shown in Figure 3 and eFigure 4. Models adding social media use are shown in eFigure 6 and eFigure 7. So that nested models are directly comparable, both models were fit on a common complete-case sample per outcome, defined by complete data on every candidate covariate including political affiliation (childhood vaccination, n = 30,163; acetaminophen, n = 30,168). Political affiliation is not a covariate in these models; 125 respondents were excluded for missing political affiliation and none for any other covariate. Race contrasts are relative to White respondents.

© 2026 Perlis RH et al. *JAMA Health Forum*.

eFigure 1. State-weighted endorsement of acetaminophen in pregnancy as an autism risk factor, by state.

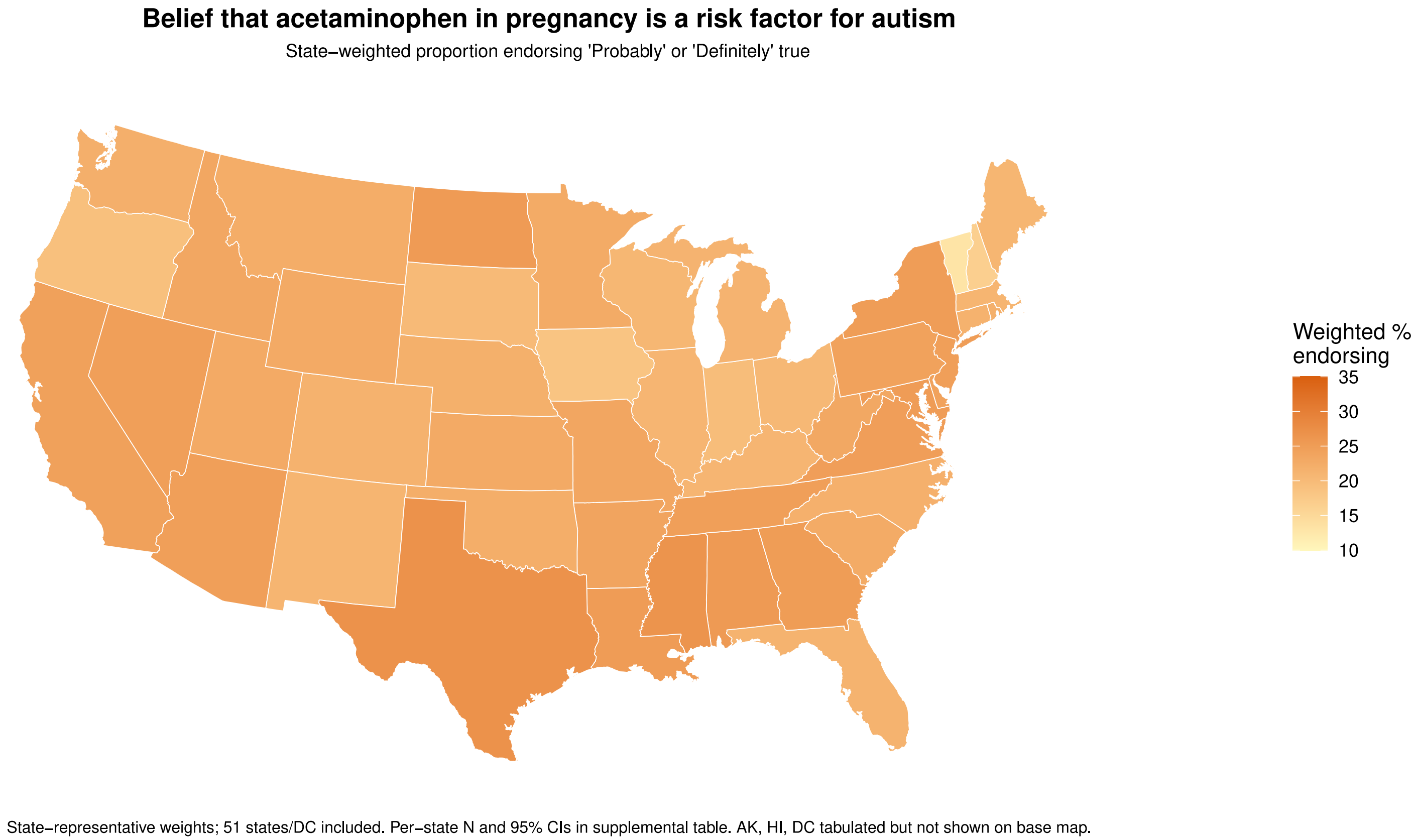

© 2026 Perlis RH et al. *JAMA Health Forum*.

eFigure 2. State-weighted endorsement of genetic factors as an autism risk factor, by state.

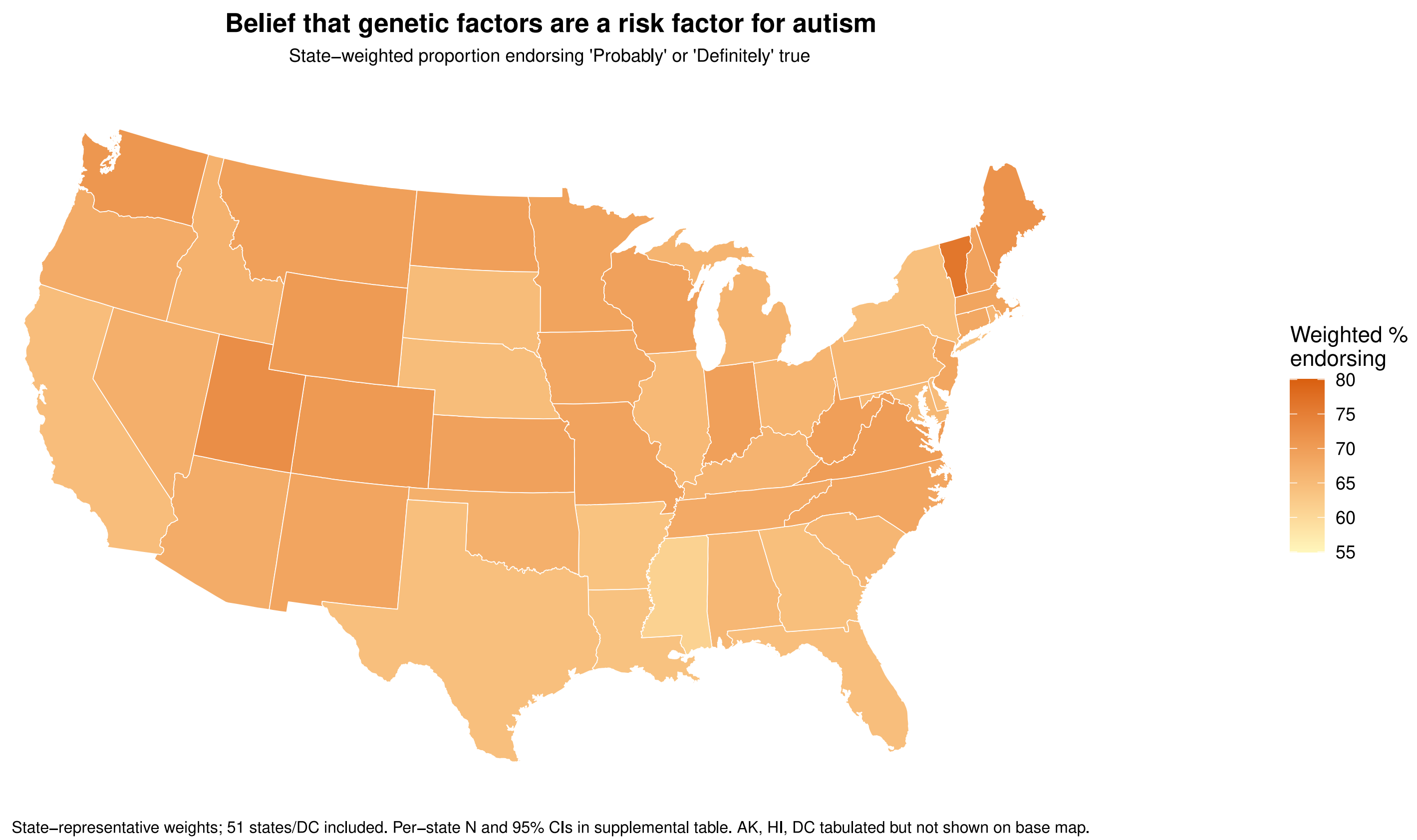

© 2026 Perlis RH et al. *JAMA Health Forum*.

eTable 3. Per-state unweighted sample size and state-weighted proportion endorsing each belief (95% CI)

State	No.	Childhood vaccination, % (95% CI)	Acetaminophen, % (95% CI)	Genetic factors, % (95% CI)
Alabama	569	28.5 (24.6-32.7)	25.4 (21.7-29.5)	65.6 (61.2-69.7)
Alaska	360	33.8 (28.3-39.9)	32.0 (26.6-38.0)	70.7 (65.1-75.8)
Arizona	588	27.5 (23.7-31.8)	24.7 (20.9-28.8)	67.5 (63.0-71.6)
Arkansas	414	27.1 (22.5-32.3)	23.8 (19.2-29.1)	63.7 (58.1-69.0)
California	1699	26.2 (23.9-28.6)	24.4 (22.1-26.7)	64.6 (61.9-67.1)
Colorado	561	24.0 (20.3-28.2)	21.5 (17.9-25.5)	70.5 (66.3-74.5)
Connecticut	423	24.0 (19.8-28.7)	21.5 (17.6-26.1)	68.1 (63.1-72.8)
Delaware	427	24.9 (20.7-29.6)	24.2 (20.1-28.9)	64.9 (59.3-70.1)
District of Columbia	386	28.5 (23.4-34.3)	29.3 (24.0-35.2)	70.6 (64.6-76.0)
Florida	1228	31.2 (28.4-34.2)	21.4 (18.9-24.0)	64.4 (61.3-67.4)
Georgia	839	30.6 (27.4-34.1)	25.1 (22.0-28.4)	64.4 (60.7-67.9)
Hawaii	324	22.1 (17.4-27.6)	20.6 (15.9-26.3)	59.9 (53.2-66.2)
Idaho	430	28.9 (24.1-34.3)	23.2 (18.8-28.3)	66.5 (60.9-71.6)
Illinois	895	24.9 (22.0-28.1)	20.9 (18.2-23.9)	65.3 (61.8-68.7)
Indiana	567	26.7 (22.9-30.8)	19.6 (16.3-23.5)	69.6 (65.3-73.5)
Iowa	453	24.4 (19.8-29.6)	18.5 (14.4-23.4)	68.3 (62.9-73.3)
Kansas	427	26.3 (21.8-31.4)	22.8 (18.6-27.6)	69.2 (64.0-74.0)
Kentucky	529	25.0 (21.3-29.2)	21.0 (17.5-24.9)	66.3 (61.6-70.6)
Louisiana	576	25.2 (21.7-29.1)	25.1 (21.5-29.0)	63.8 (59.5-67.9)
Maine	417	27.3 (22.6-32.4)	20.8 (16.7-25.6)	71.7 (66.4-76.4)
Maryland	595	24.5 (20.9-28.6)	25.1 (21.4-29.2)	65.6 (61.2-69.7)
Massachusetts	457	25.2 (21.0-30.0)	21.0 (17.1-25.5)	68.7 (63.7-73.3)
Michigan	771	25.9 (22.8-29.4)	21.2 (18.3-24.5)	66.2 (62.5-69.7)
Minnesota	549	25.8 (21.8-30.2)	22.6 (19.0-26.8)	68.8 (64.3-73.0)
Mississippi	556	30.9 (26.6-35.5)	26.5 (22.5-31.0)	61.2 (56.3-65.8)
Missouri	600	26.4 (22.7-30.5)	23.5 (19.9-27.6)	68.9 (64.7-72.9)
Montana	403	27.7 (22.7-33.4)	22.3 (17.7-27.7)	69.5 (63.8-74.7)
Nebraska	504	23.0 (18.6-28.0)	21.9 (17.5-27.0)	64.6 (59.2-69.7)
Nevada	532	27.4 (23.3-31.9)	24.6 (20.7-29.1)	67.0 (62.3-71.3)
New Hampshire	461	23.8 (19.5-28.7)	16.6 (12.9-21.0)	69.1 (63.9-73.9)
New Jersey	608	29.5 (25.7-33.5)	24.7 (21.2-28.6)	68.6 (64.4-72.4)
New Mexico	402	26.9 (22.0-32.5)	21.0 (16.4-26.5)	68.6 (62.7-74.0)
New York	1325	28.9 (26.1-31.8)	25.0 (22.4-27.7)	64.1 (60.9-67.2)
North Carolina	764	24.9 (21.8-28.3)	21.7 (18.7-25.1)	68.4 (64.8-71.8)
North Dakota	343	32.7 (26.8-39.3)	25.3 (19.6-32.0)	69.7 (63.6-75.2)
Ohio	848	24.6 (21.4-28.1)	20.4 (17.3-23.8)	66.0 (62.2-69.6)
Oklahoma	517	27.7 (23.8-32.1)	22.2 (18.6-26.3)	66.9 (62.4-71.1)
Oregon	477	25.1 (21.0-29.6)	19.2 (15.7-23.4)	67.6 (62.8-72.1)
Pennsylvania	797	24.4 (21.3-27.8)	24.2 (21.0-27.7)	65.9 (62.1-69.5)
Rhode Island	325	26.2 (21.0-32.2)	21.9 (17.1-27.6)	65.8 (59.6-71.4)
South Carolina	470	27.2 (22.9-31.9)	22.7 (18.7-27.3)	65.8 (60.8-70.4)
South Dakota	417	26.7 (21.9-32.0)	20.1 (15.8-25.3)	64.8 (59.3-70.0)
Tennessee	591	27.0 (23.4-31.0)	24.6 (21.1-28.5)	67.8 (63.7-71.6)

© 2026 Perlis RH et al. *JAMA Health Forum*.

Texas	1519	29.1 (26.7-31.7)	26.9 (24.5-29.4)	64.3 (61.6-66.9)
Utah	388	25.8 (21.2-31.1)	22.8 (18.3-28.1)	72.5 (66.9-77.4)
Vermont	328	20.6 (15.8-26.4)	13.1 (9.4-18.1)	76.3 (70.4-81.4)
Virginia	693	29.4 (25.9-33.3)	24.7 (21.4-28.4)	70.0 (66.2-73.6)
Washington	642	24.8 (21.4-28.5)	22.0 (18.8-25.6)	71.0 (67.0-74.6)
West Virginia	467	28.0 (23.3-33.3)	23.2 (18.8-28.2)	69.7 (63.8-74.9)
Wisconsin	563	22.3 (18.7-26.4)	20.7 (17.2-24.8)	69.2 (64.9-73.3)
Wyoming	364	24.8 (19.9-30.4)	22.8 (17.9-28.7)	70.4 (64.2-75.9)

State-representative survey weights applied; 95% CIs computed on the logit scale. No. is the unweighted number of respondents per state.

© 2026 Perlis RH et al. *JAMA Health Forum*.

eFigure 3. Multivariable model for endorsement of childhood vaccination as an autism risk factor, adding political affiliation.

Model 1 + Party: Sociodemographic Features Associated with Vaccine−Autism Belief

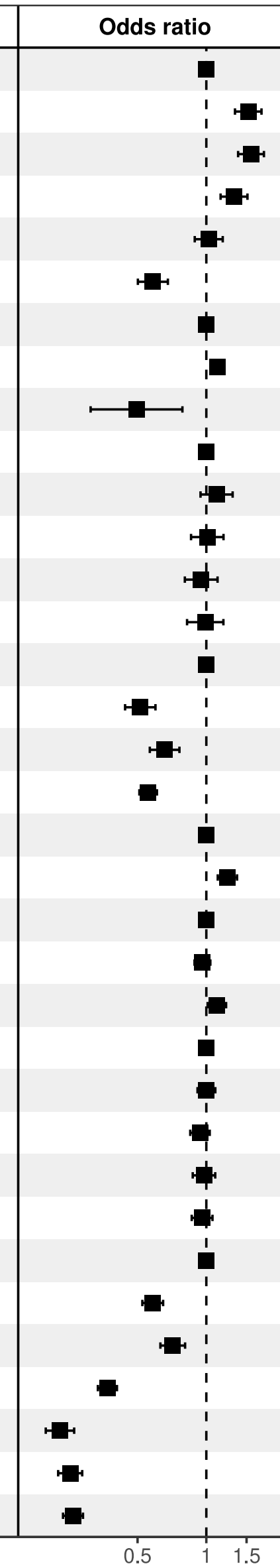

Variable		N	Odds ratio	p
**Age**	18 to 24	2261	Reference	
	25 to 34	5446	1.53 (1.33, 1.74)	<0.001
	35 to 44	6979	1.57 (1.38, 1.79)	<0.001
	45 to 54	5747	1.32 (1.15, 1.51)	<0.001
	55 to 64	4759	1.02 (0.89, 1.18)	0.736
	65 and over	4971	0.58 (0.50, 0.68)	<0.001
**Gender**	Female	16321	Reference	
	Male	13650	1.12 (1.05, 1.19)	<0.001
	Nonbinary	192	0.49 (0.31, 0.79)	0.003
**Education**	Some High School or Less	1315	Reference	
	High School Graduate	8718	1.11 (0.94, 1.30)	0.210
	Some College	7351	1.01 (0.86, 1.19)	0.909
	College Degree	9573	0.95 (0.81, 1.12)	0.538
	Graduate Degree	3206	0.99 (0.82, 1.19)	0.905
**Race**	African American	5426	Reference	
	Asian American	1715	0.51 (0.44, 0.60)	<0.001
	Other	2047	0.66 (0.57, 0.76)	<0.001
	White	20975	0.56 (0.51, 0.61)	<0.001
**Ethnicity**	Not Hispanic	26048	Reference	
	Hispanic	4115	1.24 (1.12, 1.36)	<0.001
**Urbanicity**	Rural	5959	Reference	
	Suburban	16109	0.96 (0.89, 1.04)	0.334
	Urban	8095	1.11 (1.01, 1.22)	0.026
**Household Income**	<$25K	7409	Reference	
	$25K−<$50K	7654	1.00 (0.92, 1.09)	0.985
	$50K−<$75K	5781	0.94 (0.85, 1.03)	0.201
	$75K−<$100K	3685	0.98 (0.87, 1.09)	0.684
	$100K+	5634	0.96 (0.86, 1.06)	0.424
**Political Affiliation**	Strong Republican	5990	Reference	
	Republican	3563	0.58 (0.52, 0.65)	<0.001
	Lean Republican	2061	0.71 (0.63, 0.81)	<0.001
	Independent	6420	0.37 (0.34, 0.41)	<0.001
	Lean Democrat	2365	0.23 (0.20, 0.26)	<0.001
	Democrat	3529	0.25 (0.22, 0.29)	<0.001
	Strong Democrat	6235	0.26 (0.24, 0.29)	<0.001

© 2026 Perlis RH et al. *JAMA Health Forum*.

eFigure 4. Multivariable model for endorsement of acetaminophen in pregnancy as an autism risk factor.

Model 1: Sociodemographic Features Associated with Acetaminophen−Autism Belief

Variable		N	Odds ratio	p
**Age**	18 to 24	2261	Reference	
	25 to 34	5444	1.14 (1.00, 1.30)	0.044
	35 to 44	6990	1.05 (0.93, 1.19)	0.451
	45 to 54	5756	0.77 (0.67, 0.87)	<0.001
	55 to 64	4750	0.62 (0.54, 0.72)	<0.001
	65 and over	4967	0.43 (0.37, 0.50)	<0.001
**Gender**	Female	16328	Reference	
	Male	13647	1.39 (1.30, 1.48)	<0.001
	Nonbinary	193	0.30 (0.17, 0.52)	<0.001
**Education**	Some High School or Less	1315	Reference	
	High School Graduate	8721	1.05 (0.89, 1.24)	0.561
	Some College	7355	0.87 (0.74, 1.03)	0.115
	College Degree	9572	0.94 (0.80, 1.12)	0.513
	Graduate Degree	3205	1.13 (0.93, 1.36)	0.210
**Race**	African American	5430	Reference	
	Asian American	1715	0.58 (0.50, 0.68)	<0.001
	Other	2052	0.76 (0.66, 0.88)	<0.001
	White	20971	0.80 (0.73, 0.86)	<0.001
**Ethnicity**	Not Hispanic	26051	Reference	
	Hispanic	4117	1.16 (1.05, 1.28)	0.003
**Urbanicity**	Rural	5965	Reference	
	Suburban	16111	0.86 (0.79, 0.94)	<0.001
	Urban	8092	1.03 (0.93, 1.13)	0.559
**Household Income**	<$25K	7410	Reference	
	$25K−<$50K	7657	0.96 (0.87, 1.05)	0.363
	$50K−<$75K	5779	0.90 (0.82, 1.00)	0.056
	$75K−<$100K	3687	1.03 (0.92, 1.15)	0.643
	$100K+	5635	1.13 (1.01, 1.26)	0.028

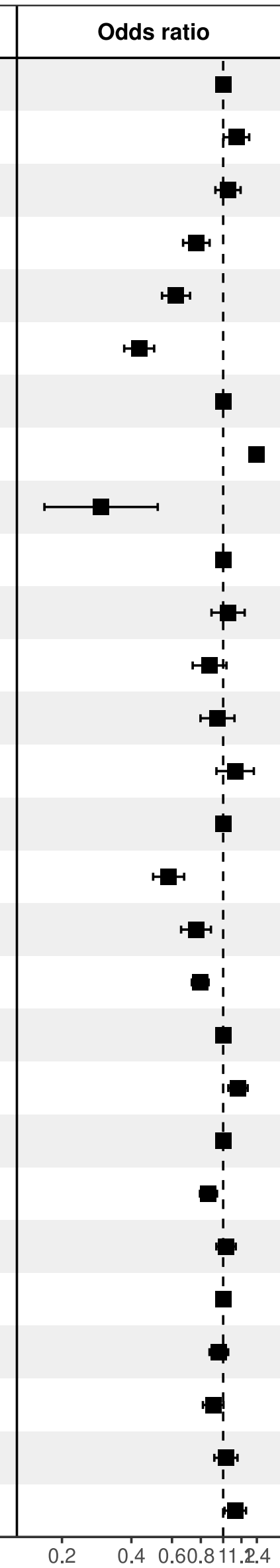

© 2026 Perlis RH et al. *JAMA Health Forum*.

eFigure 5. Multivariable model for endorsement of acetaminophen in pregnancy, adding political affiliation.

Features Associated with Acetaminophen−Autism Belief (with Party)

Variable		N	Odds ratio	p
**Age**	18 to 24	2261	Reference	
	25 to 34	5444	1.07 (0.94, 1.23)	0.288
	35 to 44	6990	0.97 (0.85, 1.11)	0.655
	45 to 54	5756	0.71 (0.62, 0.82)	<0.001
	55 to 64	4750	0.56 (0.49, 0.65)	<0.001
	65 and over	4967	0.39 (0.33, 0.45)	<0.001
**Gender**	Female	16328	Reference	
	Male	13647	1.23 (1.15, 1.31)	<0.001
	Nonbinary	193	0.38 (0.22, 0.67)	<0.001
**Education**	Some High School or Less	1315	Reference	
	High School Graduate	8721	1.04 (0.88, 1.23)	0.644
	Some College	7355	0.89 (0.75, 1.06)	0.199
	College Degree	9572	0.99 (0.83, 1.17)	0.882
	Graduate Degree	3205	1.16 (0.95, 1.40)	0.140
**Race**	African American	5430	Reference	
	Asian American	1715	0.51 (0.44, 0.60)	<0.001
	Other	2052	0.65 (0.56, 0.76)	<0.001
	White	20971	0.58 (0.53, 0.64)	<0.001
**Ethnicity**	Not Hispanic	26051	Reference	
	Hispanic	4117	1.25 (1.13, 1.38)	<0.001
**Urbanicity**	Rural	5965	Reference	
	Suburban	16111	0.92 (0.84, 1.00)	0.047
	Urban	8092	1.11 (1.00, 1.23)	0.041
**Household Income**	<$25K	7410	Reference	
	$25K−<$50K	7657	0.91 (0.83, 1.00)	0.062
	$50K−<$75K	5779	0.84 (0.76, 0.93)	0.001
	$75K−<$100K	3687	0.94 (0.83, 1.06)	0.289
	$100K+	5635	0.98 (0.87, 1.09)	0.689
**Social Media Use**	Less than weekly	5547	Reference	
	Weekly to daily	10633	0.98 (0.89, 1.07)	0.645
	Multiple times daily	13988	0.82 (0.75, 0.90)	<0.001
**Political Affiliation**	Strong Republican	5993	Reference	
	Republican	3563	0.59 (0.53, 0.66)	<0.001
	Lean Republican	2056	0.67 (0.59, 0.77)	<0.001
	Independent	6425	0.36 (0.33, 0.40)	<0.001
	Lean Democrat	2368	0.26 (0.22, 0.30)	<0.001
	Democrat	3532	0.33 (0.29, 0.38)	<0.001
	Strong Democrat	6231	0.27 (0.25, 0.30)	<0.001

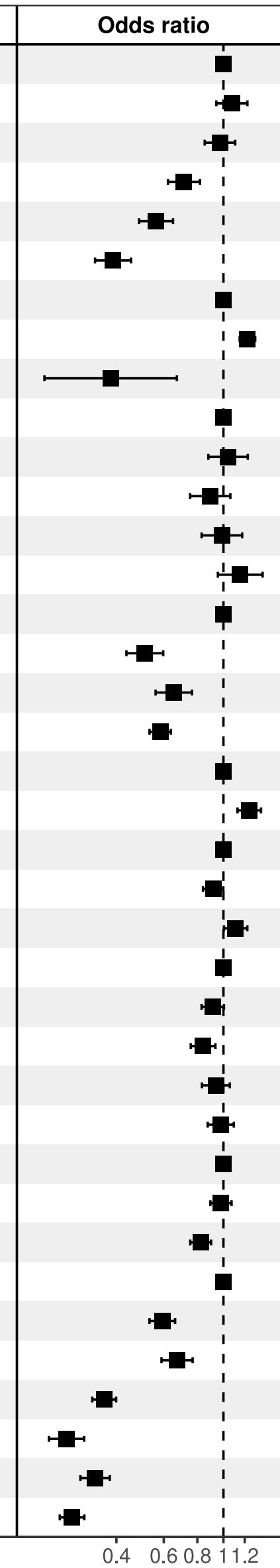

© 2026 Perlis RH et al. *JAMA Health Forum*.

eFigure 6. Social media use and endorsement of childhood vaccination as an autism risk factor.

Model 2: With Social Media Use

Variable		N	Odds ratio	p
**Age**	18 to 24	2261	Reference	
	25 to 34	5446	1.54 (1.35, 1.76)	<0.001
	35 to 44	6979	1.60 (1.41, 1.82)	<0.001
	45 to 54	5747	1.34 (1.18, 1.53)	<0.001
	55 to 64	4759	1.09 (0.95, 1.25)	0.221
	65 and over	4971	0.62 (0.54, 0.72)	<0.001
**Gender**	Female	16321	Reference	
	Male	13650	1.23 (1.16, 1.31)	<0.001
	Nonbinary	192	0.40 (0.25, 0.64)	<0.001
**Education**	Some High School or Less	1315	Reference	
	High School Graduate	8718	1.11 (0.95, 1.29)	0.201
	Some College	7351	1.01 (0.86, 1.19)	0.887
	College Degree	9573	0.93 (0.79, 1.09)	0.347
	Graduate Degree	3206	0.98 (0.82, 1.16)	0.784
**Race**	African American	5426	Reference	
	Asian American	1715	0.61 (0.53, 0.71)	<0.001
	Other	2047	0.80 (0.70, 0.93)	0.003
	White	20975	0.81 (0.75, 0.88)	<0.001
**Ethnicity**	Not Hispanic	26048	Reference	
	Hispanic	4115	1.11 (1.01, 1.22)	0.027
**Urbanicity**	Rural	5959	Reference	
	Suburban	16109	0.89 (0.82, 0.96)	0.004
	Urban	8095	1.00 (0.91, 1.09)	0.945
**Household Income**	<$25K	7409	Reference	
	$25K–<$50K	7654	1.05 (0.96, 1.15)	0.273
	$50K–<$75K	5781	1.02 (0.93, 1.12)	0.664
	$75K–<$100K	3685	1.09 (0.98, 1.22)	0.113
	$100K+	5634	1.13 (1.02, 1.25)	0.023
**Social Media Use**	Less than weekly	5549	Reference	
	Weekly to daily	10620	0.87 (0.80, 0.95)	0.001
	Multiple times daily	13994	0.72 (0.66, 0.78)	<0.001

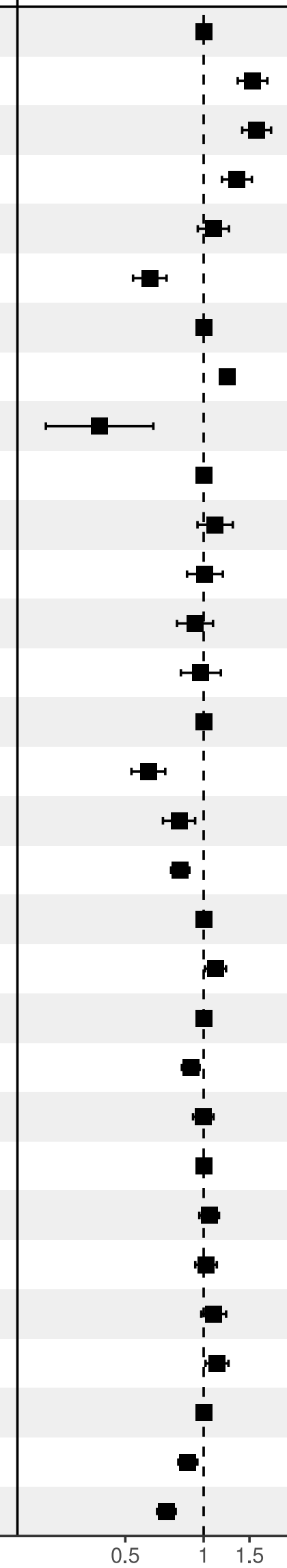

© 2026 Perlis RH et al. *JAMA Health Forum*.

eFigure 7. Social media use and endorsement of acetaminophen in pregnancy as an autism risk factor.

Model 2: Features Associated with Acetaminophen−Autism Belief, with Social Media Use

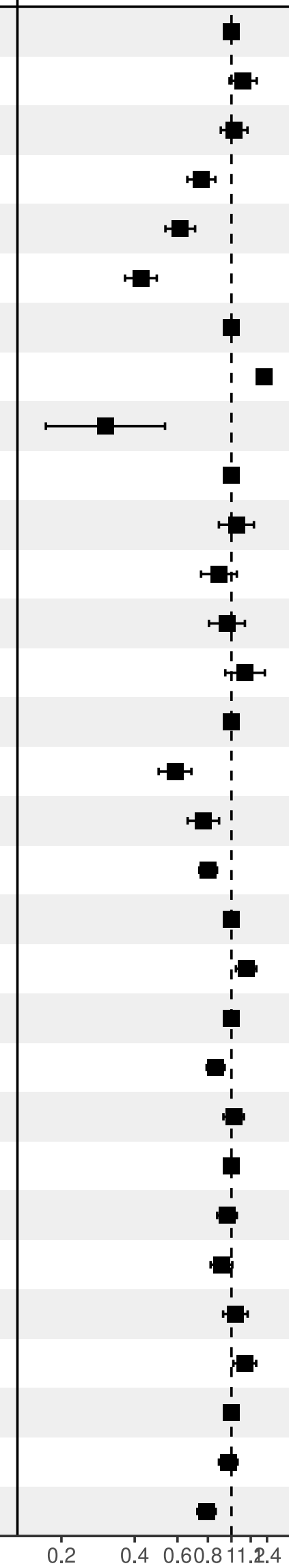

Variable		N	Odds ratio		p
**Age**	18 to 24	2261		Reference	
	25 to 34	5444		1.12 (0.98, 1.27)	0.092
	35 to 44	6990		1.03 (0.90, 1.16)	0.696
	45 to 54	5756		0.75 (0.66, 0.86)	<0.001
	55 to 64	4750		0.62 (0.54, 0.71)	<0.001
	65 and over	4967		0.42 (0.37, 0.49)	<0.001
**Gender**	Female	16328		Reference	
	Male	13647		1.36 (1.28, 1.45)	<0.001
	Nonbinary	193		0.30 (0.17, 0.53)	<0.001
**Education**	Some High School or Less	1315		Reference	
	High School Graduate	8721		1.05 (0.89, 1.24)	0.580
	Some College	7355		0.89 (0.75, 1.05)	0.170
	College Degree	9572		0.96 (0.81, 1.14)	0.620
	Graduate Degree	3205		1.14 (0.94, 1.37)	0.178
**Race**	African American	5430		Reference	
	Asian American	1715		0.59 (0.50, 0.68)	<0.001
	Other	2052		0.77 (0.66, 0.89)	<0.001
	White	20971		0.80 (0.74, 0.87)	<0.001
**Ethnicity**	Not Hispanic	26051		Reference	
	Hispanic	4117		1.15 (1.04, 1.27)	0.005
**Urbanicity**	Rural	5965		Reference	
	Suburban	16111		0.86 (0.79, 0.94)	<0.001
	Urban	8092		1.02 (0.93, 1.13)	0.673
**Household Income**	<$25K	7410		Reference	
	$25K−<$50K	7657		0.96 (0.87, 1.05)	0.372
	$50K−<$75K	5779		0.91 (0.82, 1.01)	0.070
	$75K−<$100K	3687		1.04 (0.92, 1.17)	0.531
	$100K+	5635		1.13 (1.02, 1.26)	0.022
**Social Media Use**	Less than weekly	5547		Reference	
	Weekly to daily	10633		0.97 (0.89, 1.06)	0.496
	Multiple times daily	13988		0.79 (0.72, 0.86)	<0.001

© 2026 Perlis RH et al. *JAMA Health Forum*.

eFigure 8. Trust in institutions and endorsement of acetaminophen in pregnancy as an autism risk factor.

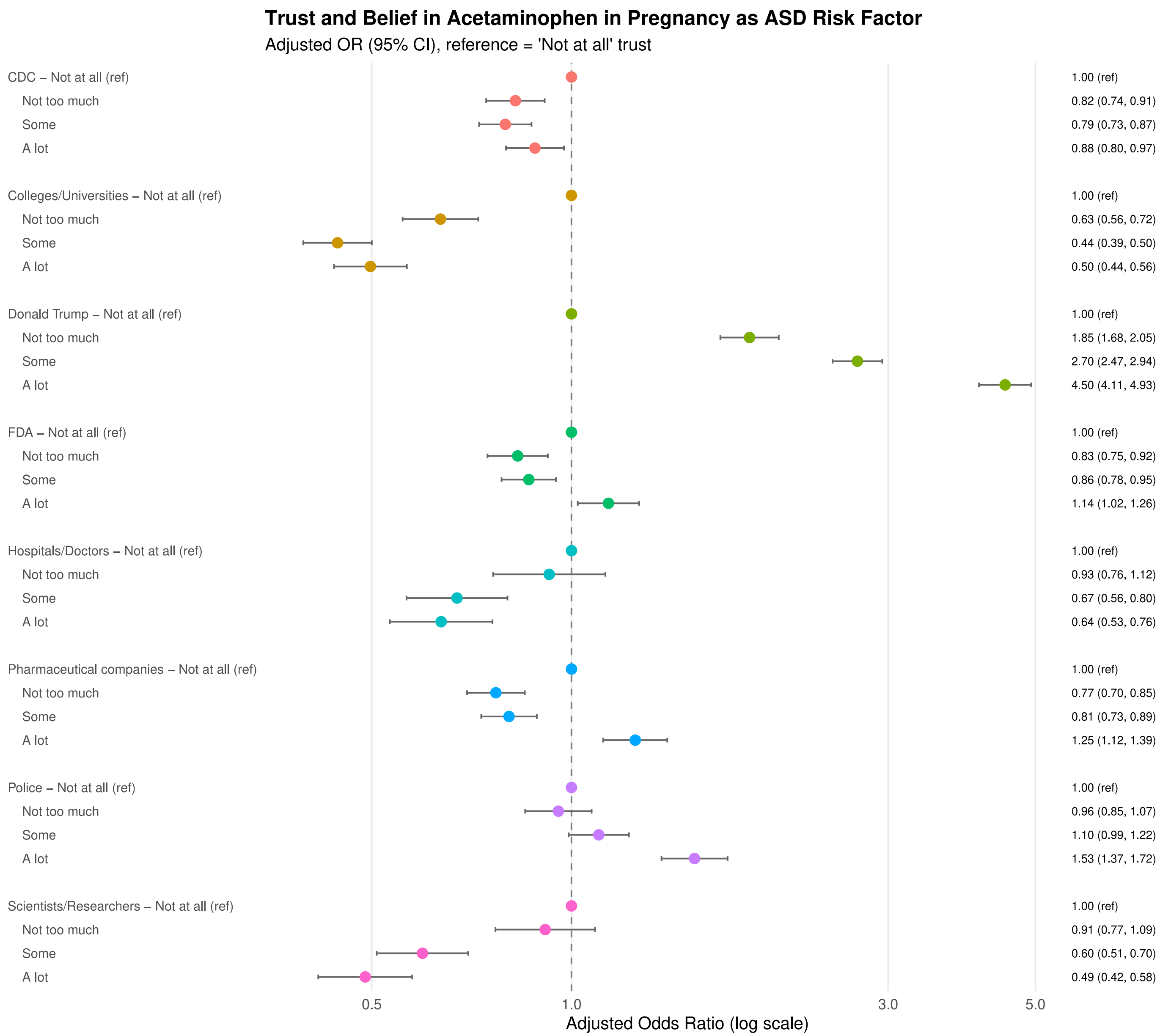

© 2026 Perlis RH et al. *JAMA Health Forum*.

eFigure 9. Trust in institutions and endorsement of genetic factors as an autism risk factor.

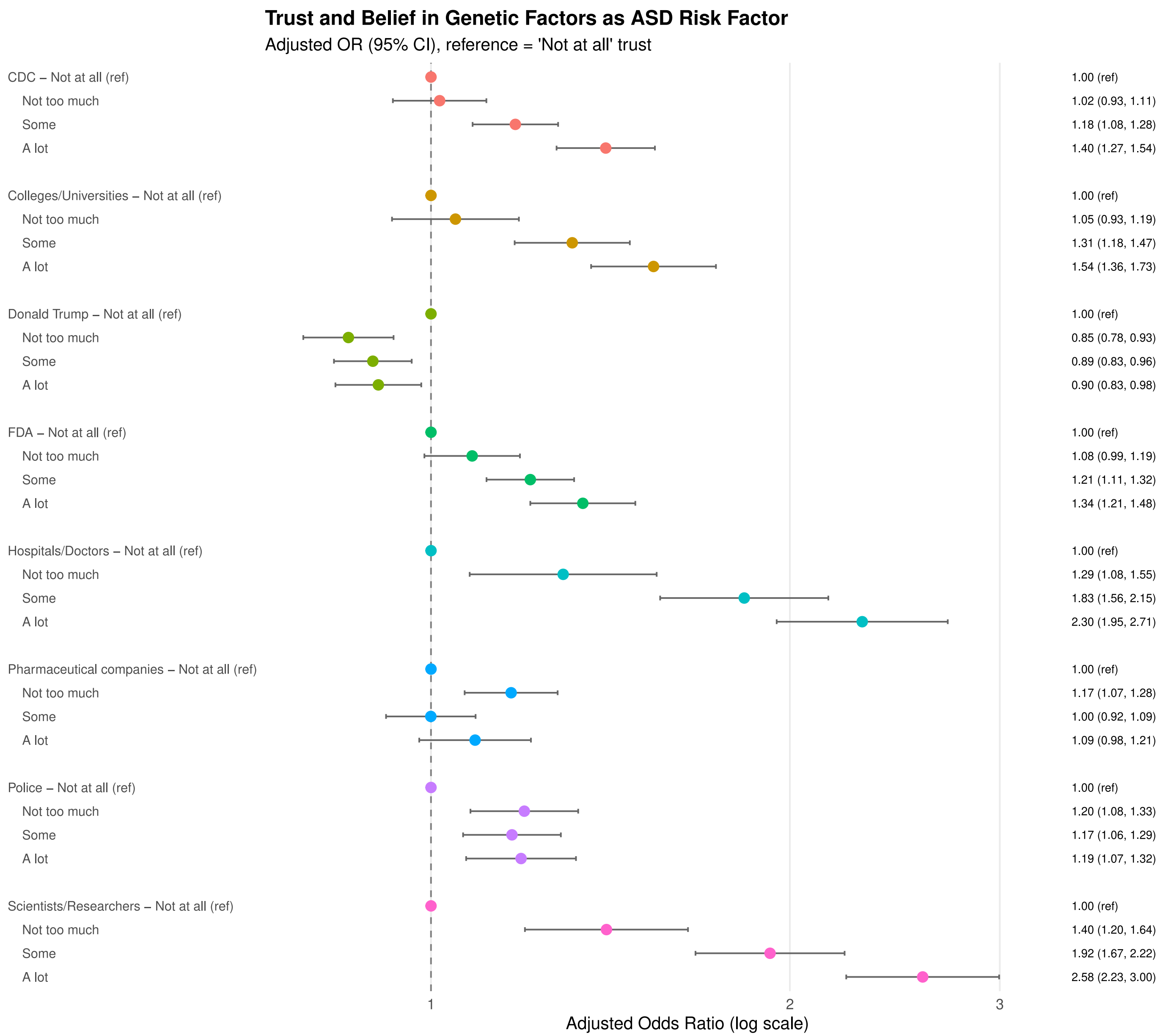

© 2026 Perlis RH et al. *JAMA Health Forum*.

eMethods 2. Principal components analysis of the twelve autism risk-factor belief items

To assess whether endorsement of the twelve autism risk-factor items reflects a single general tendency or multiple distinct dimensions, we conducted an exploratory principal components analysis.

Each item was scored on an ordinal scale from Definitely false (1) to Definitely true (5), with 'Not sure' treated as a distinct middle category (3) in the primary analysis and, in a sensitivity analysis, excluded as missing (a 4-point scale). Because the items are ordinal rather than continuous, we computed a survey-weighted polychoric correlation matrix using the national weights and extracted components from that matrix.

The number of components to retain was determined by a scree plot and parallel analysis with the effective sample size (eFigure 10); three components were retained. We applied oblique (promax) rotation. Component scores were computed for respondents with complete data on all twelve items (n = 29,604) as weighted z-scored items multiplied by the promax scoring weights.

The three retained components accounted for 67.4% of the variance after promax rotation: a general component (RC1) accounting for 40.7% of the variance and loading on the non-evidence-based and disputed items, as well as on maternal infection, the sole evidence-based item in that set (eTable 4); a second component (RC2) accounting for 11.8% and loading on evidence-based biological causes (genetic factors, prematurity, and maternal infection, which also loads on the first component); and a third component (RC3) accounting for 14.9% and loading on paternal age and male sex. Before rotation the first component accounted for 46.2% of the variance. Inter-component correlations were modest (RC1-RC2 phi = 0.25), indicating the components as distinct rather than a single relabeled factor.

We then treated each retained component score as an outcome, regressing it on the full sociodemographic covariate set, on political affiliation, and separately on trust in each of the eight named institutions and individuals, using survey weights. Trust contrasts ('A lot' vs 'Not at all') are summarized in eTable 5.

© 2026 Perlis RH et al. *JAMA Health Forum*.

eFigure 10. Scree plot and parallel analysis for the principal components analysis of the twelve belief items.

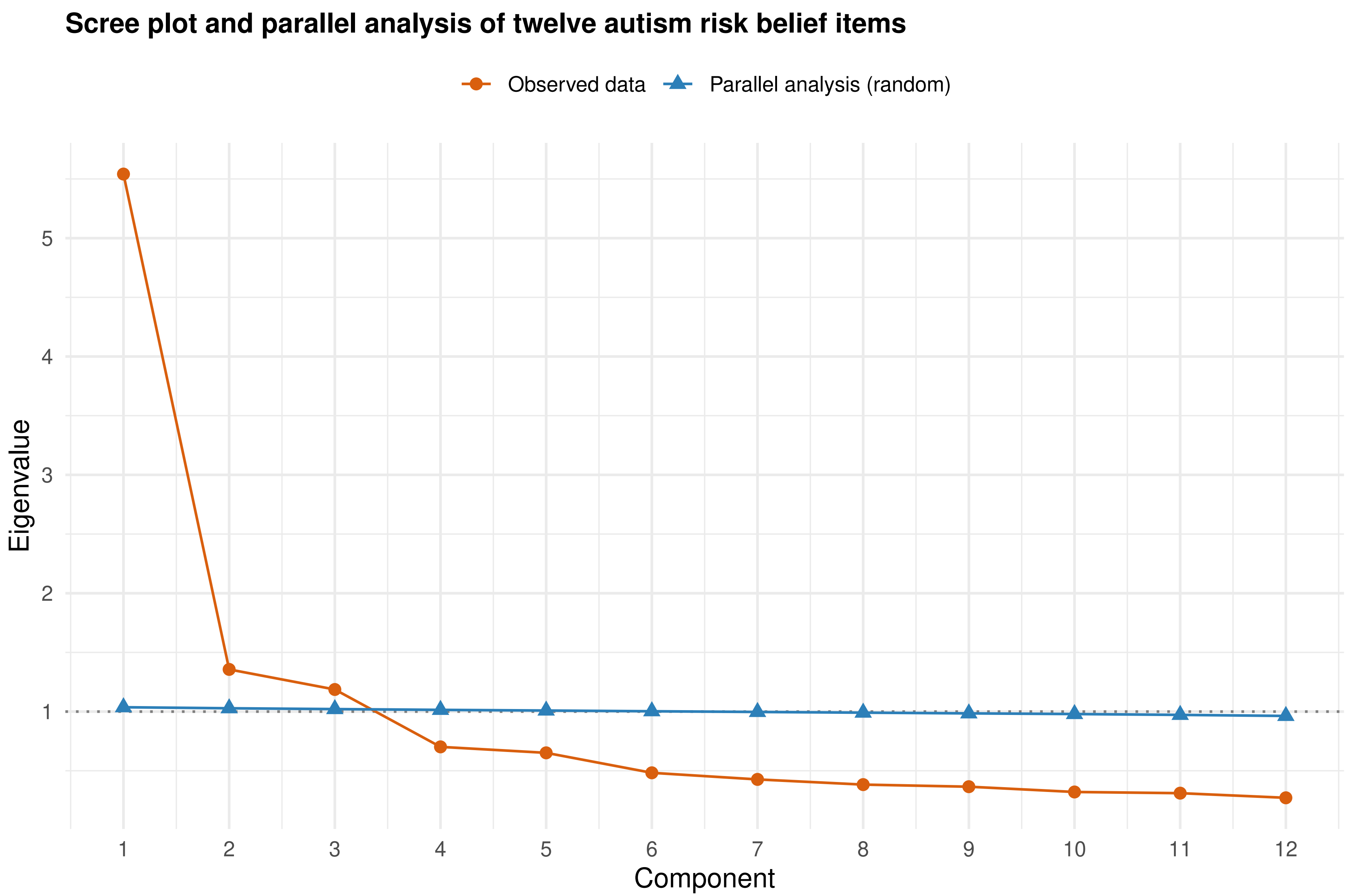

© 2026 Perlis RH et al. *JAMA Health Forum*.

eTable 4. Survey-weighted promax-rotated principal component loadings for the twelve belief items

Item	RC1 (general non-evidence)	RC2 (evidence-based)	RC3 (paternal age/male sex)	Evidence class
Genetic factors	-0.269	0.848	0.13	Evidence-based
Childhood vaccinations	0.751	-0.176	0.085	Non-evidence-based
Older father	-0.034	0.204	0.846	Evidence-based
Male sex	-0.022	0.011	0.885	Evidence-based
Artificial dyes	0.707	-0.102	0.189	Non-evidence-based
Raw fish	0.672	-0.146	0.236	Non-evidence-based
Fluoridated water	0.791	-0.182	0.13	Non-evidence-based
Premature birth	0.318	0.52	0.208	Evidence-based
Acetaminophen in pregnancy	0.836	0.007	-0.061	Non-evidence-based
Antidepressants in pregnancy	0.83	0.243	-0.202	Disputed
Maternal infections	0.638	0.462	-0.135	Evidence-based
TDaP vaccine in pregnancy	0.879	0.005	-0.147	Non-evidence-based

Loadings from a survey-weighted polychoric-correlation PCA with promax (oblique) rotation; three components retained by parallel analysis, accounting for 67.4% of variance (RC1 40.7%, RC3 14.9%, RC2 11.8%). Items scored 1=Definitely false to 5=Definitely true, with Not sure as the middle category.

© 2026 Perlis RH et al. *JAMA Health Forum*.

eTable 5. Association of trust ('A lot' vs 'Not at all') with each component score, survey-weighted, adjusted for sociodemographic covariates

Component	Institution / individual	Difference in component score (95% CI)	P value
RC1 (general non-evidence)	FDA	-0.04 (-0.09 to 0.00)	0.07
RC1 (general non-evidence)	CDC	-0.17 (-0.22 to -0.13)	<.001
RC1 (general non-evidence)	Donald Trump	0.83 (0.80 to 0.86)	<.001
RC1 (general non-evidence)	Hospitals and doctors	-0.46 (-0.55 to -0.37)	<.001
RC1 (general non-evidence)	Pharmaceutical companies	0.00 (-0.05 to 0.05)	0.97
RC1 (general non-evidence)	Scientists and researchers	-0.63 (-0.70 to -0.55)	<.001
RC1 (general non-evidence)	Colleges and universities	-0.53 (-0.58 to -0.47)	<.001
RC1 (general non-evidence)	Police	0.21 (0.16 to 0.26)	<.001
RC3 (paternal age/male sex)	FDA	0.02 (-0.03 to 0.07)	0.35
RC3 (paternal age/male sex)	CDC	-0.04 (-0.08 to 0.01)	0.13
RC3 (paternal age/male sex)	Donald Trump	0.26 (0.22 to 0.30)	<.001
RC3 (paternal age/male sex)	Hospitals and doctors	-0.14 (-0.22 to -0.06)	<.001
RC3 (paternal age/male sex)	Pharmaceutical companies	0.10 (0.05 to 0.15)	<.001
RC3 (paternal age/male sex)	Scientists and researchers	-0.19 (-0.26 to -0.12)	<.001
RC3 (paternal age/male sex)	Colleges and universities	-0.14 (-0.20 to -0.09)	<.001
RC3 (paternal age/male sex)	Police	0.08 (0.03 to 0.13)	0.003
RC2 (evidence-based)	FDA	0.20 (0.16 to 0.25)	<.001
RC2 (evidence-based)	CDC	0.22 (0.18 to 0.27)	<.001
RC2 (evidence-based)	Donald Trump	0.07 (0.03 to 0.11)	<.001
RC2 (evidence-based)	Hospitals and doctors	0.31 (0.22 to 0.41)	<.001
RC2 (evidence-based)	Pharmaceutical companies	0.09 (0.04 to 0.14)	<.001
RC2 (evidence-based)	Scientists and researchers	0.35 (0.27 to 0.44)	<.001
RC2 (evidence-based)	Colleges and universities	0.19 (0.13 to 0.25)	<.001
RC2 (evidence-based)	Police	0.09 (0.03 to 0.14)	0.001

Each row is the 'A lot' vs 'Not at all' trust contrast from a separate survey-weighted linear model of the component score adjusted for age, gender, education, income, race, ethnicity, and urbanicity. Positive values indicate higher component scores.

© 2026 Perlis RH et al. *JAMA Health Forum*.

Trust in doctors/scientists lowers the general non-evidence component (RC1) but raises the evidence-based component (RC2), inconsistent with a single acquiescence factor.

© 2026 Perlis RH et al. *JAMA Health Forum*.
