# Supplementary material for: Knowledge and Misinformation Regarding Autism Risk Factors in a Survey of US Adults
Source: JAMA Health Forum. 2026 Nov 6;7(11):e264105. doi: 10.1001/jamahealthforum.2026.4105 (PMC13744024; doi:10.1001/jamahealthforum.2026.4105)
Supplement: Supplementary file 2 [file jamahealthforum-e264105-s002.pdf]

Data Sharing Statement

Perlis. Knowledge and Misinformation Regarding Autism Risk Factors in a Survey of US Adults. *JAMA Health Forum*. Published November 06, 2026.
doi:10.1001/jamahealthforum.2026.4105

Data
Data available: No
